# Investigating the Ligand‐Binding Properties of N‐arylbenzimidazoles as Novel Elastase Inhibitors

**DOI:** 10.1002/cmdc.202500879

**Published:** 2025-12-21

**Authors:** Giovanna Pitasi, Sonia Floris, Francesca Mancuso, Giulia Savoca, Rosaria Gitto, Antonella Fais, Laura De Luca

**Affiliations:** ^1^ Department of Chemical, Biological, Pharmaceutical and Environmental Sciences University of Messina Viale F. Stagno D’Alcontres 31 I‐98125 Messina Italy; ^2^ Department of Life and Environment Sciences University of Cagliari I‐09042 Monserrato Cagliari Italy

**Keywords:** elastases, molecular docking, N1‐arylmethylbenzimidazole scaffolds, syntheses

## Abstract

Human elastase 1 has been shown to possess an important role in maintaining skin stability and elasticity through the proteolytic cleavage of elastin (ELN), a hydrophobic protein that serves as a key component of extracellular matrix in the skin. The development of antielastase agents represents a promising therapeutic approach for treating skin pathologies characterized by elastin degradation, with applications in both dermatology and cosmetology. Reversible inhibitors represent a therapeutic strategy, offering selective inhibition of elastase proteolytic activity while preserving the function of other physiologically essential serine proteases. Using porcine pancreatic elastase (PPE) as a well‐established surrogate of human skin elastase, a focused series of noncovalent inhibitors designed to bind the catalytic area of PPE is assayed. Several compounds display an antielastase activity, including *N*‐(2‐bromophenyl)‐2‐(6‐chloro‐1‐(3,5‐dimethylbenzyl)‐1*H*‐benzo[d]imidazol‐2‐ylthio)acetamide (**7**) that exhibits the most potent inhibitory effects (IC_50_ = 41.1 µM), similar to standard compound oleanolic acid (IC_50_ value of 25.7 µM). The observed structure–activity relationship is further validated through molecular docking and dynamic studies, which provide mechanistic understanding of the binding interactions and establish suggestions for further rational drug design.

## Graphical abstract

A series of novel N‐[(3,5‐dimethylphenyl)methyl]‐1H‐benzimidazole derivatives is designed, synthesized, and evaluated for inhibitory activity against elastase. Several compounds demonstrate an antielastase activity, including the N‐(2‐bromophenyl)‐2‐(6‐chloro‐1‐(3,5‐dimethylbenzyl)‐1H‐benzo[d]imidazol‐2‐ylthio)acetamide, showing the greatest potency (IC_50_ = 41.1 µM) through a competitive inhibition mechanism. Computational analyses elucidate the binding modes of these derivatives and provide valuable insights to guide future rational drug design efforts.

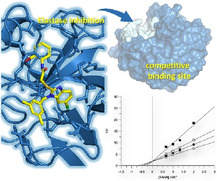

## 1 Introduction

The human skin exerts an important role of the primary barrier against pathological factors promoting aging processes that lead to wrinkles, laxity, roughness, and loss of elasticity. The skin is composed of three distinct layers classified into epidermis, dermis, and subcutaneous tissues. In more detail, the dermis contains several proteins, including collagen, elastin, and proteoglycans.^[^ ^1^ ^]^ Among them, collagen and elastin (ELN) are crucial components of the extracellular matrix in the skin; they are responsible for structural stability, keeping the skin elastic and hydrated. During aging, the skin reduces the epidermis layer, its hydration, through the loss of collagen and elastic fibers. Moreover, increased elastase activity and subsequent degradation of elastin fibers are related to several skin disorders (psoriasis, contact dermatitis, and atopic dermatitis).^[^ ^2^ ^,^ ^3^ ^]^

Six distinct genes are responsible for encoding human elastases 1, 2, 2A, 2B, 3A, and 3B. Among them, the elastase 1 (EC 3.4.21.36) is expressed in skin keratinocytes, whereas the human neutrophil elastase (EC 3.4.21.37, HNE, elastase 2 or leukocyte elastase) secreted by neutrophils plays a relevant role in the immune response toward bacterial infections, as well as inflammation and tissue remodeling.^[^ ^4^ ^,^ ^5^ ^]^ The HNE is considered responsible for elastin degradation and other extracellular matrix components of endothelial tissues that generate active fragments, inducing inflammatory and immune responses. The inhibition of HNE has been investigated for the therapeutic management of arthritis, cystic fibrosis, respiratory diseases, and cancer.^[^ ^6^, ^7^, ^8^, ^9^ ^]^ In the case of the elastase 1, the proteolytic action toward elastin impairs the skin structure. Due to the role that elastase 1 covers in this proteolytic process, various investigations have been carried out to discover inhibitors as useful agents for the treatment of skin pathologies in distinct areas of skincare solutions covering dermatology and cosmetology.^[^ ^10^ ^]^ Additionally, elastase inhibitors might be useful to fight the virulence of microorganisms responsible for several skin infective pathologies.

Considerable attention has been devoted to understanding the binding mechanisms of inhibitory compounds targeting elastase enzymes in various organisms; structural insights were achieved through investigating the binding interactions with the analogous enzyme porcine pancreatic elastase (PPE)^[^ ^11^, ^12^, ^13^ ^]^ that is currently used as the poor, expensive isozyme for preliminary biochemical assays. There is a high degree of sequence identity shared by PPE and pancreatic elastases from other species. Although PPE shares only about 40% sequence homology with HNE at the primary structure level, their tertiary structures are remarkably alike, particularly in the region surrounding the catalytic site.^[^ ^11^, ^12^, ^13^ ^]^

Numerous elastase inhibitors from synthetic^[^ ^14^ ^]^ and natural sources^[^ ^15^ ^,^ ^16^ ^]^ have been discovered. A broad range of HNE inhibitors has emerged from medicinal chemistry investigations that have been classified according to their mechanism of action or binding site as reversible or irreversible inhibitors, as well as competitive or noncompetitive inhibitors.^[^ ^7^ ^,^ ^17^, ^18^, ^19^ ^]^ Numerous inhibitors based on peptide and non‐peptide scaffolds^[^ ^20^ ^]^ exhibit irreversible binding properties through covalent modification of critical residues in the catalytic cleft.

Particularly, the most popular elastase inhibitors currently employed in therapy are characterized by a peptide chemical scaffold mimicking the endogenous substrate.^[^ ^21^ ^]^ However, they often suffer from the lack of selectivity toward other serine proteases.

**Figure 1:**
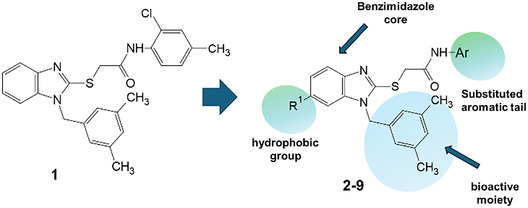
Chemical structures of N1‐arylmethylbenzimidazole derivative **1** and designed analog compounds [(3,5‐dimethylphenyl)methyl]‐1*H*‐benzimidazoles (**2**–**9**).

A recent multistep computational investigation carried out by our group led to the identification of the N1‐arylmethylbenzimidazole derivative **1** (CAS 1,562,665‐34‐2, *N*‐(2‐chloro‐4‐methylphenyl)‐2‐[[1‐[(3,5‐dimethylphenyl)methyl]‐1*H*‐benzimidazol‐2‐yl]thio]acetamide, **Figure** **1**) as a new chemotype demonstrating the ability to inhibit PPE at micromolar concentration (IC_50_ value of 60.4 ± 1.98 µM).^[^ ^22^ ^]^

This compound possessed chemical and structural properties that allowed it to establish profitable interactions with PPE active site being a reversible competitive inhibitor.^[^ ^22^ ^]^ Building on the promising inhibitory effects of prototype compound **1**, we report here a focused collection of its eight analog derivatives **2–9** (Figure 1) that have been investigated to highlight a preliminary structure–activity relationship (SAR) analysis for this class of competitive PPE inhibitors being the N1‐arylmethylbenzimidazole scaffold.

These new derivatives were designed by simple structural modifications of parent compound 1, such as introducing a chlorine atom at C‐6 (R_1_) of benzene‐fused ring and changing the substituents at 2‐ and/or 4‐position of aromatic ring (Ar moiety in Figure 1) linked to nitrogen atom of the thioacetamide connecting moiety. The obtained SAR data, combined with molecular docking and dynamic studies, could represent a contribution toward the identification of new reversible and selective antielastase agents as skincare agents.

## 2 Results and Discussion

### 2.1 Synthetic Efforts

**Scheme 1:**
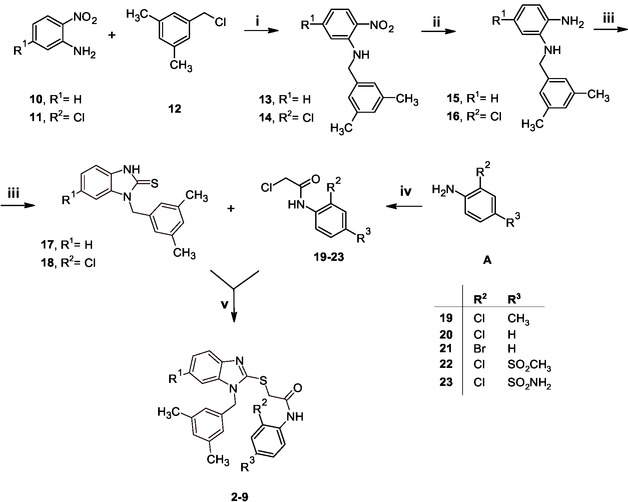
Reagents and conditions: (i) H_2_O, reflux, 3 h; (ii) NH_2_‐NH_2_ H_2_O, 10% Pd/C, EtOH, 80 °C, 1 h; (iii) TCDI, pyridine, rt, 1 h; (iv) chloroacetylchloride, DIPEA, DCM, rt, 1 h; (v) K_2_CO_3_, DMF, rt, 2 h.

**Scheme** **1** outlined the multistep route to synthesize desired compounds **2**–**9** that were prepared according to our previously reported procedures^[^ ^23^ ^,^ ^24^ ^]^ by introducing few slight modifications as detailed below.

First, the 2‐nitroaniline derivative (**10** or **11**) reacted with 3,5‐dimethylbenzyl bromide (**12**) to give corresponding *N*‐alkyl derivatives **13**–**14**; unlike previously reported procedures,^[^ ^23^ ^,^ ^24^ ^]^ we employed water as the sole solvent.^[^ ^25^ ^]^ Although the above‐mentioned conditions resulted in slightly reduced yields when compared to the previously reported synthetic conditions in *N,N*‐dimethylformamide, we chose to apply this greener and more environmentally friendly alternative. Small modifications were also made to this first step in terms of the stoichiometric ratios of the reagents, since it was observed that an excess of 3,5‐dimethylbenzyl bromide led to the formation of undesired side products. The *N*‐(3,5‐dimethylbenzyl)‐substituted nitroaniline intermediates **13**–**14** were subsequently reduced using hydrazine hydrate (NH_2_‐NH_2_ H_2_O) in the presence of 10% Pd/C as catalyst.^[^ ^26^ ^]^ This catalytic transfer hydrogenation method advanced efficiently, affording the corresponding *o*‐phenylenediamine derivatives **15**–**16** in quantitative yields, so that the crude intermediates were isolated by simple crystallization, without the need for further purification. Then, the cyclization of the amine derivatives **15**–**16** was efficiently accomplished by treatment with thiocarbonyldiimidazole (TCDI), which quickly and completely reacted to furnish the targeted benzimidazole compounds **17**–**18**. The suitable commercially available anilines (**A**) reacted with chloroacetyl chloride at room temperature under basic conditions to give **19**–**23**. In the last step of synthetic route, the benzimidazole compounds **17**–**18** were alkylated by reaction with **19**–**23** to give the desired *N*‐aryl‐thiobenzimidazoles **2**–**9**, which were obtained in excellent yields after purification by either crystallization from ethanol or flash column chromatography on silica gel. All final products **2**–**9** were thoroughly characterized using ^1^H NMR spectroscopy, with spectra matching data that were previously reported for these compounds.^[^ ^23^ ^,^ ^24^ ^]^ Moreover, ^13^C NMR analysis was conducted to provide additional structural elucidation as detailed in the Experimental Section. All ^1^H NMR and ^13^C NMR spectra of final compounds **2**–**9** are reported in the Supporting Information (Figure S1‐S20, Supporting Information).

In addition, we assessed the relative lipophilicity of the investigated compounds using the reverse‐phase thin layer chromatography (RP‐TLC), following the procedure detailed in Experimental Section. Based on retention factor (*R*_F_), we obtained the corresponding *R*_M_ values for the homogenous series of compounds **1**–**9**. The applied methodology is described in detail in the Experimental Section. The *R*_M_ measurements revealed that all compounds possessed *R*_M_ values ranging from 0.550 to 1.221, with high values obtained for compounds **6**–**8**, whereas the 4‐sulfamoyl‐substituted compound **5** possessed lower lipophilicity with respect to the corresponding unsubstituted analog compound **2**; these data were in good agreement with the predicted values calculated with Absorption, Distribution, Metabolism, and Excretion (ADME) Percepta platform (see Table 2 in Experimental Section).

### 2.2 Elastase Inhibition and Structure–Activity Relationship (SAR) Analysis

Compounds **2**–**9** were tested for their ability to inhibit PPE, employing *N*‐succ‐(Ala)3‐nitroanilide (SANA) as the substrate, in accordance with the methodology previously reported^[^ ^27^ ^]^ and oleanolic acid as reference compound.^[^ ^27^ ^]^ In this preliminary assay, we determined the percentage of inhibition at fixed dose of 50 µM of tested compound, and the results are collected in **Table** **1** in comparison with previously reported lead compound **1**. For this small series of compounds, the deletion of 4^′^‐methyl‐substituent on aromatic ring of prototype **1** did not influence the inhibitory effects, so the unsubstituted compounds **2** and **3** demonstrated the same activity as parent methyl‐substituted compound **1**; as expected, the introduction of polar substituents (e.g. SO_2_CH_3_ or SO_2_NH_2_) reduced the ability to inhibit PPE. Notably, all compounds bearing a chlorine atom at C‐6 position (as R_1_ substituent in compounds **6**–**9**) exhibited improved inhibitory effects when compared to unsubstituted analog compounds **1**–**4** (R_1_ = H). The most interesting compounds were derivatives **6** and **7** bearing a halogen atom at C‐2^′^‐position on the phenyl ring of the benzamide moiety; they displayed IC_50_ values of 44.8 ± 1.4 and 41.1 ± 1.9 µM, respectively, thus proving promising activity when compared with standard compound oleanolic acid >(IC_50_ value of 25.7 ± 1.38 µM).

**Table 1:** Percentage of inhibition (I%) at 50 µM against PPE for benzimidazole derivatives **1–9**.

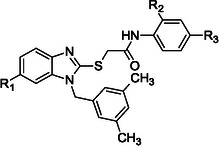					
Compounds	R_1_	R_2_	R_3_	%I @50 μMa)	IC_50_ [μM]
**1** a)	‐H	Cl	CH_3_	30.0 ± 2.1	60.4 ± 2.0b)
**2**	‐H	Cl	H	29.1 ± 2.3	N.D.
**3**	‐H	Br	H	26.5 ± 0.4	N.D.
**4**	‐H	Cl	SO_2_CH_3_	17.3 ± 0.7	N.D.
**5**	‐H	Cl	SO_2_NH_2_	19.3 ± 0.9	N.D.
**6**	‐Cl	Cl	H	57.4 ± 2.6	44.8 ± 1.4
**7**	‐Cl	Br	H	62.2 ± 2.1	41.1 ± 1.9
**8**	‐Cl	Cl	CH_3_	40.6 ± 0.7	N.D.
**9**	‐Cl	Cl	SO_2_CH_3_	31.0 ± 0.6	N.D.
Oleanolic acid					25.7 ± 1.38

a) I% values represent the mean ± standard deviation for three independent measurements (*n* = 3).

b) Data taken from ref. [22]. N.D. not determined.

**Figure 2:**
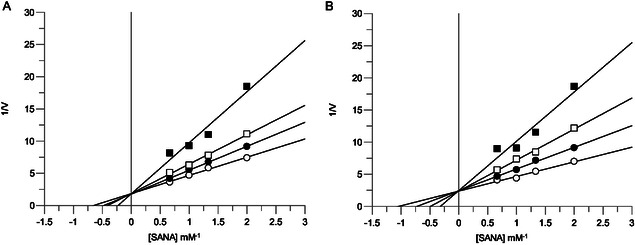
Inhibition of PPE activity by compound A) **6** and B) **7**. Lineweaver–Burk plot for inhibition of PPE activity using SANA as a substrate at different concentrations (0.5, 0.75, 1.0, and 1.5 mM). Concentrations of each compound were: 0 (○), 10 μM (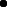), 25 μM (□), and 50 μM (▪).

**Figure 3:**
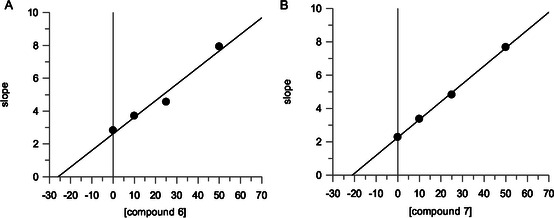
The secondary plot of slope (Km/Vmax) versus concentrations of compound A) **6** and B) **7** to determine the inhibition constant (K_i_).

Kinetic characterization was performed to determine how compounds **6** and **7** interact with PPE. The inhibition mechanism was assessed using Lineweaver–Burk plot analysis. Plots of the initial rates of PPE activity in the presence of increasing substrate concentrations yielded, for compounds **6** and **7**, a family of straight lines with different slopes but intersecting each other on the *Y*‐axis (**Figure** **2**A‐B), thus suggesting competitive inhibitory effects. The inhibition constant (K_i_) for the inhibitors binding with the free enzyme (E) was obtained from the secondary plot as 25.8 µM (compound **6**) and 21 µM (compound **7**) (**Figure** **3**A‐B).

### 2.3 In Silico Studies

To investigate the binding mode of the new series of eight benzimidazole‐based compounds (**2**–**9**), we performed docking studies using the PPE crystal structure (PDB code: 1ELE) with the GOLD program (v2024),^[^ ^28^ ^]^ following the same protocol described in our previous paper^[^ ^22^ ^]^ for parent compound **1**. Then, the resulting protein–ligand complexes were subjected to molecular dynamics simulations using the Desmond module within the Schrödinger suite.^[^ ^29^ ^]^ Computational studies revealed that studied compounds demonstrated the ability to occupy the PPE cavity in the same region as compound **1**, as shown in Figure S21, Supporting Information.

**Figure 4:**
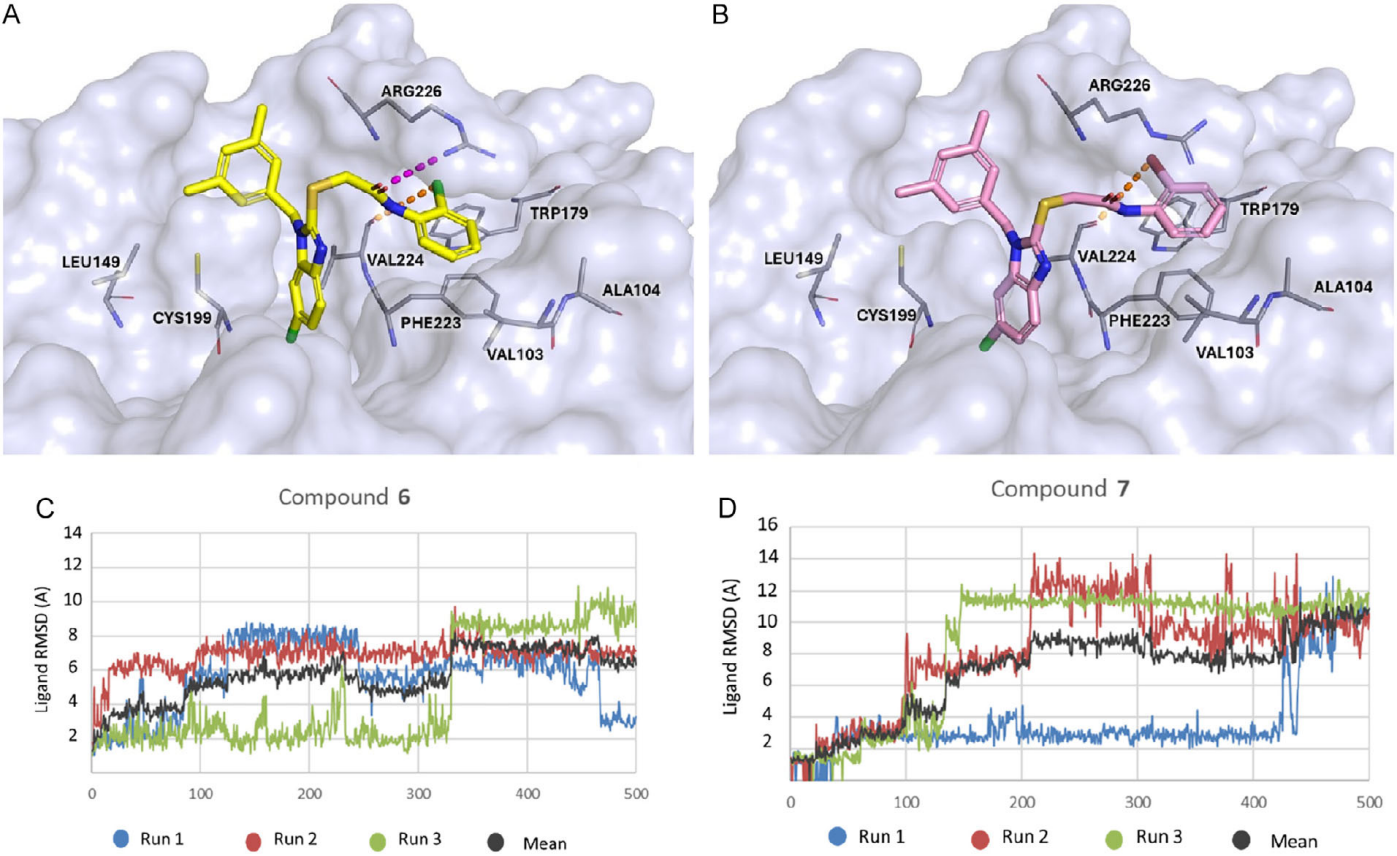
Plausible binding mode of compound **6** (yellow sticks, panel A) and compound **7** (pink sticks, panel B) within the cavity of elastase protein (PDB code: 1ELE). The protein is shown as a light‐blue surface, with amino acid residues represented as light‐blue sticks. Hydrogen bonds and halogen bonds are indicated by magenta and orange dashed lines, respectively. Images were generated using PyMOL (https://www.pymol.org/). The plots show the A) RMSD of compound **6** (Panel C) and compound **7** (Panel D) with respect to their initial structures over 500 ns of molecular dynamics simulations performed in triplicate. The black line represents the average RMSD across the three simulations.

**Figure 5:**
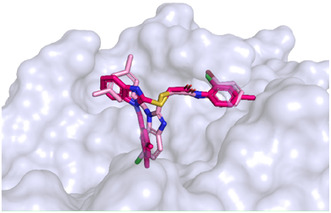
Superimposition of the reference compound **1** (hotpink sticks) with the most active chlorinated derivative, compound **7** (pink sticks). The protein (PDB code 1ELE) is represented as a light blue surface, and amino acid residues are represented as light blue sticks. Images were created using PyMOL (https://www.pymol.org/).

Given the superior inhibitory activity of compounds **6** and **7**, we concentrated on detailing their binding modes (**Figure** **4**A‐B). The interaction analysis revealed a conserved binding mode, with both compounds engaging in key stabilizing hydrophobic contacts with residues Phe223, Val224, and hydrogen bond with Arg226. Moreover, both compounds **6** and **7** established a halogen bond between their halogen substituent (chlorine atom in compound **6** and bromine atom in compound **7**) and Val224, further reinforcing their anchoring within the binding cavity as displayed in **Figure** **5**E,F for **6** and **7**, respectively. To complement these observations, MM‐GBSA binding free energy calculations^[^ ^30^ ^]^ were performed on the protein–ligand complexes obtained from molecular docking. The predicted binding free energies were –111.38 kcal mol^−1^ for compound **6** and –112.34 kcal mol^−1^ for compound **7**, thus indicating highly favorable and similar interaction strengths. The binding energy values were found to be consistent with the experimental inhibitory activity data for active compounds **6** **–7** when compared with other studied inhibitors (Table S1, Supporting Information).

Molecular dynamics simulations, performed in three independent replicas, were carried out to assess the stability of the compounds **6** and **7** within the binding pocket (Figure 4C–D). The root‐mean‐square deviation (RMSD) plots showed that both ligands reached a maximum deviation of ≈12 Å, indicating a certain degree of conformational flexibility inside the cavity. Despite these fluctuations, the trajectories revealed that the ligands remained stably accommodated throughout the simulation time, maintaining interactions with the key residues. As shown in Figure 4C–D, the three replicas exhibited comparable RMSD trends, and the overall mean trajectory (black line) reflects a consistent behavior across simulations. This agreement among replicas confirms the reproducibility of the results and suggests that the observed fluctuations primarily reflect intrinsic ligand mobility and local conformational adjustments within the binding pocket, rather than any instability (separate RMSD plots for each replica are reported in the Supporting Information).

Furthermore, to gain further insights into the binding modes of derivatives **6** and **7**, we compared them with reference ligand **1**. Based on the consideration that derivatives **6** and **7** shared the same binding mode, the superimposition of the binding pose of the derivative **7** and parent compound **1** is displayed in Figure 5. It was possible to note that these two compounds assumed a different orientation within the active site: the chlorinated (R_1_ = Cl*)* benzimidazole moiety of compound **7** occupied the subpocket previously engaged by the dimethylbenzyl group of reference compound **1**, while the dimethylbenzyl group of compound **7** shifted toward the region occupied by the benzimidazole core of parent compound **1**. This apparent “binding mode inversion” suggested that the chlorine substituent (R_1_ = Cl) on the benzimidazole ring may stabilize an alternative and more favorable ligand orientation within the active site.

Finally, for compounds **6** and **7,** we conducted an additional in silico ADME profiling to assess skin permeability, skin sensitization, and mutagenicity through Ames test, using the pkCSM freeware software^[^ ^31^ ^]^ (https://biosig.lab.uq.edu.au/pkcsm/prediction, accessed on July 27, 2025). Furthermore, the presence of PAINS (Pan Assay Interference Compounds) and Brenk structural alerts was evaluated using webtool SwissADME^[^ ^32^ ^]^ (http://www.swissadme.ch/, accessed on July 28, 2025). Both compounds showed acceptable skin permeability values (–3.207 for compound **6** and –2.777 for compound **7**) and were predicted to be nonsensitizing to skin and nonmutagenic. Furthermore, SwissADME analysis revealed no PAINS or Brenk structural alerts for both compounds.

## 3 Conclusion

Taken together, these results underlined that for the studied *N*‐[(3,5‐dimethylphenyl)methyl]‐1*H*‐benzimidazoles (**2**–**9**), we found a positive influence of C‐6 chlorine substitution on PPE inhibitory potency in agreement with a more effective binding orientation within the hydrophobic area of catalytic cavity. Our computational studies revealed the consistent tendency across molecular docking, MD simulations, and MM‐GBSA binding free energy calculations, thus validating the potential of compounds **6**–**7** as new lead candidates bearing more lipophilic moieties when compared to parent compound **1**. This achievement was also supported by the enzymatic data, which revealed enhanced inhibitory potency and confirmed a competitive type of PPE inhibition.

## 4 Experimental Section

### Chemistry

All reagents and solvents were purchased from “ThermoFisher Scientific‐Alfa Aesar” (Segrate, Italy) and “Merck Sigma Aldrich” (Milano, Italy). The thin layer chromatography (TLC) and RP‐TLC were performed with precoated silica gel plates (glass sheets) with fluorescent indicator F254 (Merck, 60, F254). Nuclear magnetic resonance (NMR) spectra (^1^H and ^13^C NMR) were recorded on Varian Gemini 500 (Palo Alto, CA, USA) in DMSO‐d6. For target compounds **2–9**, the NMR chemical shifts (*δ*) were reported in parts per million (ppm) and coupling constants (J) in Hertz (Hz). Melting points were recorded on Buchi B‐545 (BUCHI Labortechnik AG, Flawil, Switzerland) and were uncorrected. The purity of final compounds **2–9** was observed to exceed ≥95% by elemental analyses (C, H, N)recorded with “Carlo Erba 1106 Analyzer” instrument, so that the found values were within ±0.4% of the calculated values.

### General Procedure to Synthesize the *N*‐Substituted‐2‐Nitroaniline Intermediates 13–14

A mixture of the commercially available nitroaniline derivative **10** or **11** (2.9 mmol) and 3,5‐dimethylbenzyl bromide (**12**, 692.2 mg, 3.48 mmol) in H_2_O (5 mL) was stirred for 100 °C for 1.5 h;^[^ ^25^ ^]^ the mixture was then cooled to room temperature and treated with a saturated NaHCO_3_ aqueous solution; subsequently, the mixture was extracted with EtOAc and dried over Na_2_SO_4_; after removal of the solvent under reduced pressure, the residue was crystallized from EtOH to furnish corresponding intermediates **13**–**14** in 45–50% yields. The intermediates **13**–**14** were preliminarily characterized, thus matching with the compounds reported in previous papers.

*N*‐[(3,5‐Dimethylphenyl)methyl]‐2‐nitroaniline (**13**)^[^ ^23^ ^]^

5‐Chloro‐*N*‐[(3,5‐dimethylphenyl)methyl]‐2‐nitroaniline (**14**)^[^ ^33^ ^]^

### General Procedure to Synthesize the *o*‐Phenylenediamine Intermediates 15–16

The obtained *N*‐substituted‐2‐nitroaniline **13** or **14** (1 mmol) reacted with NH_2_‐NH_2_ H_2_O (10 mmol) in EtOH (15 mL) at 80 °C for 1 h in the presence of a catalytic amount of 10% Pd/C; after completion of nitroreduction, the mixture was cooled, filtered by celite cake, and washed with EtOAc; the reaction mixture was evaporated under vacuum and the residue was treated with ethanol to give desired amine derivative **15** or **16** in good yields (>95%), with no further purification needed. The obtained intermediates **15** and **16** were preliminarily characterized, thus matching with the compounds reported in previous papers.

2‐Amino‐1‐(3,5‐dimethylbenzyl)‐aniline (**15**)^[^ ^23^ ^]^

2‐Amino‐5‐chloro‐1‐(3,5‐dimethylbenzyl)‐aniline (**16**)^[^ ^33^ ^]^

### General Procedure to Synthesize the 1‐(3,5‐dimethylbenzyl)‐1,3‐dihydro‐2*H*‐benzimidazole‐2‐thione Intermediates 17–18

To a solution of *o*‐phenylenediamine derivative **15** or **16** (1 mmol) in pyridine (10 mL), the 1,1^′^‐thiocarbonyldiimidazole (250 mg, 1.4 mmol) was added and the resulting mixture was stirred for 1h at room temperature. After this time, the mixture was quenched with H_2_O and the precipitate was filtered off to give the desired 1‐(3,5‐dimethylbenzyl)−1,3‐dihydro‐2*H*‐benzimidazole‐2‐thione derivative **17** or **18** in 43–60% yields; the obtained intermediates **17–18** were preliminarily characterized, thus matching with the compounds reported in previous papers.

1‐(3,5‐Dimethylbenzyl)−1,3‐dihydro‐2*H*‐benzimidazole‐2‐thione (**17**)^[^ ^23^ ^,^ ^34^ ^]^

6‐Chloro‐1‐(3,5‐dimethylbenzyl)−1,3‐dihydro‐2*H*‐benzimidazole‐2‐thione (**18**)^[^ ^35^ ^]^

### General Procedure to Synthesize the 2‐Chloro‐*N*‐Phenylacetamide Intermediates 19–23

To a mixture of commercially available anilines type **A** (1 mmol) in DCM (5 mL) and *N*,*N*‐diisopropylethylamine (1 mmol), the chloroacetyl chloride (1 mmol) was added dropwise and the resulting mixture was stirred for 1 h at room temperature; the reaction was quenched with a saturated NaHCO_3_ aqueous solution, then extracted with EtOAc, dried, and the solvent was removed under reduced pressure to furnish crude products that were crystallized from EtOH, giving target 2‐chloro‐*N*‐phenylacetamide derivatives **19**–**23** in 45–90% yields; the obtained intermediates **19–23** were preliminarily characterized, thus matching with the compounds reported in previous papers.

2‐Chloro‐*N*‐(2‐chloro‐4‐methylphenyl)acetamide (**19**)^[^ ^23^ ^]^

2‐Chloro‐*N*‐(2‐chlorophenyl)acetamide (**20**)^[^ ^23^ ^,^ ^36^ ^]^

*N*‐(2‐Bromophenyl)‐2‐chloroacetamide (**21**)^[^ ^23^ ^]^

2‐Chloro‐*N*‐[2‐chloro‐4‐(methanesulfonyl)phenyl]acetamide (**22**)^[^ ^23^ ^]^

2‐Chloro‐*N*‐(2‐chloro‐4‐sulfamoylphenyl)acetamide (**23**)^[^ ^23^ ^]^

### General Procedure to Synthesize the Target *N*‐aryl‐thiobenzimidazoles 2–9

Finally, the reaction of 1‐(3,5‐dimethylbenzyl)−1,3‐dihydro‐2*H*‐benzimidazol‐2‐one derivative **17** or **18** (1 mmol) and the appropriate 2‐chloro‐*N*‐phenylacetamide **19–23** (1 mmol) was performed in DMF (3 mL), in presence of K_2_CO_3_ (138 mg, 1 mmol) for 1h at room temperature; after this time, the mixture was quenched by the addition of saturated NaHCO_3_ aqueous solution, extracted with EtOAc, and evaporated under reduced pressure to furnish crude products; by treatment with EtOH, the desired final compounds **2**–**9** were isolated as white solid in 85–90% yields; before performing biochemical assays, the final compounds **2–9** were carefully characterized as reported below.

### *N*‐(2‐Chlorophenyl)‐2‐(1‐(3,5‐dimethylbenzyl)‐1*H*‐benzo[d]imidazol‐2‐ylthio)acetamide (2)


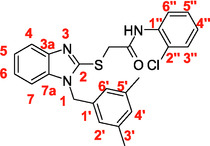


CAS Number:1,562,664‐78‐1. M.p. 134–136 °C. ^1^H NMR (500 MHz, DMSO‐*d*_6_, ppm): *δ* = 2.14 (s, 6H; ‐CH_3_), 4.32 (s, 2H; ‐SCH_2_CO‐), 5.32 (s, 2H; ‐NCH_2_Ar), 6.80 (s, 2H; Ar‐H, H‐2‘ and 6‘), 6.87 (s, 1H; Ar‐H, H‐4^′^), 7.12 (t, 3J = 7.83 Hz, 1H, H‐3’’;), 7.17–7.18 (m, 2H; Ar‐H, H‐5 and H‐6), 7.29 (t, ^*3*^ *J* = 7.8 Hz, 1H; Ar‐H, H‐4’’;), 7.42 (d, ^*3*^ *J* = 7.83 Hz; 1H, Ar‐H, H‐5’’;), 7.44 (bs, 1H; Ar‐H, H‐4), 7.56 (bs, 1H; Ar‐H, H‐7), 7.89 (d, ^*3*^ *J* = 7.8 Hz, 1H; Ar‐H, H‐6’’;), 10.17 (bs, 1H, ‐NHCO). ^13^C NMR (126 MHz, DMSO‐*d*_6_): *δ *= 21.3 (CH_3_), 36.4 (‐S*C*H_2_CO‐), 47.3 (‐NCH_2_Ar), 110.5 (C‐7), 118.1 (C‐4), 122.4 (C‐5), 122.5 (C‐6), 124.7 (C‐6’’;), 125.1 (C‐2^′^ and C‐6^′^), 125.3 (C‐5’’;),126.4 (C‐4’’;), 128.0 (C‐2’’;), 129.7 (C‐4^′^), 129.9 (C‐3’’;), 135.2 (C‐1’’;), 136.4 (C‐1^′^), 136.8 (C‐4a), 138.3 (C‐3^′^ and C‐5^′^), 143.1 (C‐3a), 151.7 (C‐2), 167.3 (CO). Elemental analysis calcd for C_24_H_22_ClN_3_OS: C 66.12, H 5.09, N 9.64, found: C 66.09, H 5.11, N 9.68.

### *N*‐(2‐Bromophenyl)‐2‐(1‐(3,5‐dimethylbenzyl)‐1*H*‐benzo[d]imidazol‐2‐ylthio)acetamide (3)


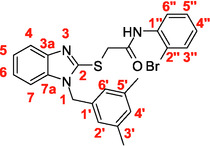


CAS Number:1,562,664‐89‐4. M.p. 115–117 °C. ^1^H NMR (500 MHz, DMSO‐*d*_6_, ppm): *δ* = 2.16 (s, 6H; ‐CH_3_), 4.32 (s, 2H; ‐SCH_2_CO‐), 5.33 (s, 2H; ‐NCH_2_Ar), 6.82 (s, 2H; Ar‐H, H‐2‘ and 6‘), 6.88 (s, 1H; Ar‐H, H‐4^′^), 7.08 (t, ^*3*^ *J* = 7.8 Hz, 1H; H‐3’’;), 7.16‐7.17 (m, 2H; Ar‐H, H‐5 and H‐6), 7.34 (t, ^*3*^ *J* = 7.8 Hz; 1H, Ar‐H, H‐4″;), 7.47–7.48 (m, 1H; Ar‐H, H‐5’’;), 7.59‐7.61 (m, 2H; Ar‐H, H‐4 and H‐7), 7.78 (d, ^*3*^ *J* = 7.8 Hz, 1H; H‐6’’;), 10.01 (br s; 1H, ‐NHCO). ^13^CNMR (126 MHz, DMSO‐*d*_6_): *δ *= 21.3 (CH_3_), 36.4 (S*C*H_2_CO), 47.3 (NCH_2_Ar), 110.4 (C‐7), 116.5 (C‐2’’;), 118.2 (C‐4), 122.3 (s, C‐5), 122.6 (C‐6), 125.2 (C‐2^′^ and C‐6^′^), 125.6 (C‐6’’;),127.1 (C‐4’’;), 128.6 (C‐5’’;),129.7 (C‐4^′^), 133.1 (C‐3’’;), 136.41 (C‐1’’;), 136.42 (C‐1^′^), 136.8 (C‐7a), 138.3 (C‐3^′^ and C‐5^′^), 143.1 (C‐3a), 151.6 (C‐2), 167.2 (CO). Elemental analysis calcd for C_24_H_22_BrN_3_OS: C 60.00, H 4.62, N 8.75, found: C 59.97, H 4.64, N 8.79.

### *N*‐(2‐Chloro‐4‐(methylsulfonyl)phenyl)‐2‐(1‐(3,5‐dimethylbenzyl)‐1*H*‐benzo[d]imidazol‐2‐ylthio)acetamide (4)


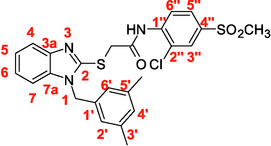


CAS Number:1,562,665‐61‐5. M.p. 212‐214 °C. ^1^H NMR (500 MHz, DMSO‐*d*_6_, ppm): *δ *= 2.15 (s, 6H; CH_3_), 3.21 (s, 3H; −SO_2_CH_3_), 4.38 (s, 2H; ‐SCH_2_CO‐), 5.33 (s, 2H; ‐NCH_2_Ar), 6.81 (s, 2H; Ar‐H, H‐2‘ and 6‘), 6.88 (s, 1H; Ar‐H, H‐4^′^), 7.17‐7.19 (m, 2H; Ar‐H, H‐5 and H‐6), 7.48–7.49 (m, 1H; Ar‐H, H‐4), 7.57‐7.59 (m, 1H; Ar‐H, H‐7), 7.84 (dd, ^*3*^ *J* = 8.31 Hz, ^*4*^ *J* = 2.0 Hz; 1H, Ar‐H, H‐5’’;), 7.98 (d, ^*4*^ *J* = 2.0 Hz; 1H, Ar‐H, H‐3’’;), 8.29 (d, ^*3*^ *J* = 8.3 Hz; 1H, Ar‐H, H‐6’’;), 10.50 (br s, 1H; ‐NHCO). ^13^C NMR (126 MHz, DMSO‐*d*_6_): *δ*= 21.3 (CH_3_), 36.5 (‐S*C*H_2_CO‐), 43.9 (SO_2_CH3), 47.4 (NCH_2_Ar), 110.5 (C‐7), 118.1 (C‐4), 122.4 (C‐5), 122.6 (C‐6), 123.6 (C‐5’’;), 124.5 (C‐6’’;), 125.2 (C‐2^′^ and C‐6^′^), 127.1 (C‐2’’;), 128.7 (C‐3’’;), 129.7 (C‐4^′^), 136.4 (C‐1^′^), 136.8 (C‐1’’;), 137.3 (C‐7a), 138.3 (C‐3^′^ and C‐5^′^), 139.8 (C‐4^′^) 143.0 (C‐3a), 151.7 (C‐2), 168.1 (CO). Elemental analysis calcd for C_25_H_24_ClN_3_O_3_S: C 58.41, H 4.71, N 8.17, found: C 58.38, H 4.73, N 8.13.

### *N*‐(2‐Chloro‐4‐sulfamoylphenyl)‐2‐(1‐(3,5‐dimethylbenzyl)‐1‐benzo[d]imidazol‐2‐ylthio)acetamide (5)


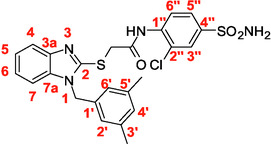


CAS Number: 1,562,665‐86‐4. M.p. 222–24 °C. ^1^H NMR (500 MHz, DMSO‐*d*_6_, ppm): *δ* = 2.16 (s, 6H, ‐CH_3_), 4.37 (s, 2H; ‐SCH_2_CO‐), 5.33 (s, 2H; ‐NCH_2_Ar), 6.81 (s, 2H; Ar‐H, H‐2‘ and 6‘), 6.85 (s, 1H; Ar‐H, H‐4^′^), 7.18‐7.19 (m, 2H; Ar‐H, H‐5 and H‐6), 7.41 (s, 2H, −SO_2_NH_2_), 7.48‐7.50 (m, 1H; Ar‐H, H‐4), 7.56–7.58 (m, 1H; Ar‐H, H‐7), 7.73 (dd, ^*3*^ *J* = 8.3 Hz, ^*4*^ *J* = 2.0 Hz; 1H, Ar‐H, H‐5’’;), 7.84 (d, ^*4*^ *J* = 2.0; 1H, Ar‐H, H‐3’’;), 8.17 (d, ^*3*^ *J* = 8.3 Hz; 1H, Ar‐H, H‐6’’;), 10.43 (br s; 1H, ‐NHCO). ^13^C NMR (126 MHz, DMSO‐*d*_6_): *δ* = 21.3 (CH_3_), 36.5 (‐S*C*H_2_CO‐), 47.4 (‐NCH_2_Ar), 110.5 (C‐7), 118.1 (C‐4), 122.4 (C‐5), 122.6 (C‐6), 123.9 (C‐5’’;), 124.5 (C‐6’’;), 125.2 (C‐6^′^), 125.6 (C‐3’’;), 127.2 (C‐2’’;), 129.7 (C‐4^′^), 136.4 (C‐1^′^), 136.8 (C‐4’’;), 138.2 (C‐7a), 138.3 (C‐3^′^ and C‐5^′^), 141.01 (C‐1’’;), 143.0 (C‐3a), 151.7 (C‐2), 167.8 (CO). Elemental analysis calcd for C_24_H_23_ClN_4_O_3_S_2_: C 55.97, H 4.50, N 10.88, found: C 56.01, H 4.47, N 10.85

### 2‐(6‐Chloro‐1‐(3,5‐dimethylbenzyl)‐1*H*‐benzo[d]imidazol‐2‐ylthio)‐*N*‐(2‐chlorophenyl)acetamide (6)


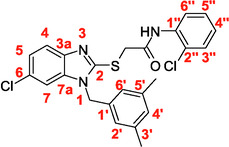


CAS Number:1,562,664‐80‐5. M.p. 182–84 °C. ^1^H NMR (500 MHz, DMSO‐*d*_6_, ppm): *δ* = 2.15 (s, 6H, ‐CH_3_), 4.34 (s, 2H, ‐SCH_2_CO‐), 5.34 (s, 2H, ‐NCH_2_Ar), 6.79 (s, 2H; Ar‐H, H‐2‘ and 6‘), 6.87 (s, 1H; Ar‐H, H‐4^′^), 7.12 (dd, ^*3*^ *J* = 7.8 Hz, ^*4*^ *J* = 1.5 Hz; 1H, Ar‐H, H‐4’’;), 7.19 (dd, ^*3*^ *J* = 8.3 Hz, ^*4*^ *J* = 2.0 Hz; 1H, Ar‐H, H‐5), 7.29 (dd, ^*3*^ *J* = 7.8 Hz, ^*4*^ *J* = 1.5 Hz, 1H, Ar‐H, H‐5’’;), 7.43 (dd, ^*3*^ *J* = 7.8 Hz, ^*4*^ *J* = 1.5 Hz; 1H, Ar‐H, H‐3’’;), 7.56 (d, ^*3*^ *J* = 8.3 Hz; 1H, Ar‐H, H‐4), 7.64 (d, ^*4*^ *J* = 2.0 Hz, 1H, Ar‐H, H‐7), 7.87 (dd, ^*3*^ *J* = 7.8 Hz, ^*4*^ *J* = 1.5 Hz; 1H, Ar‐H, H‐6’’;), 10.07 (br s, 1H, ‐NHCO). ^13^C NMR (126 MHz, DMSO‐*d*_6_): *δ* = 21.3 (CH_3_), 36.5 (‐SCH_2_CO‐), 47.4 (‐NCH_2_Ph), 110.5 (C‐7), 119.3 (C‐4), 122.6 (C‐5), 124.9 (C‐6’’;), 125.1 (C‐2^′^ and C‐6^′^), 125.5 (C‐4’’;), 126.4 (C‐6), 127.0 (C‐5’’;), 128.0 (C‐2’’;), 129.7 (C‐4^′^), 129.9 (C‐3’’;), 135.1 (C‐1’’;), 136.1 (C‐1^′^), 137.6 (C‐7a), 138.3 (C‐3^′^ and C‐5^′^), 141.9 (C‐3a), 153.3 (C‐2), 167.0 (CO). Elemental analysis calcd for C_24_H_21_Cl_2_N_3_OS: C 61.28, H 4.50, N 8.93, found: C 61.32, H 4.47, N 8.90.

### *N*‐(2‐Bromophenyl)‐2‐(6‐chloro‐1‐(3,5‐dimethylbenzyl)‐1*H*‐benzo[d]imidazol‐2‐ylthio)acetamide (7)


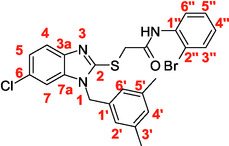


CAS Number: 1,562,664‐93‐0. M.p. 153‐55 °C. ^1^H NMR (500 MHz, DMSO‐*d*_6_, ppm): *δ* = 2.15 (s, 6H, ‐CH_3_), 4.31 (s, 2H, ‐SCH_2_CO‐), 5.33 (s, 2H, ‐NCH_2_Ar), 6.78 (s, 2H; Ar‐H, H‐2‘ and 6‘), 6.88 (s, 1H; Ar‐H, H‐4^′^), 7.07 (dd, ^*3*^ *J* = 7.8 Hz;1H, Ar‐H, H‐4’’;), 7.18 (dd, ^*3*^ *J* = 8.8 Hz, ^*4*^ *J* = 2.0 Hz; 1H, Ar‐H, H‐5), 7.33 (dd, ^*3*^ *J* = 7.8 Hz; 1H, Ar‐H, H‐5’’;), 7.55–7.60 (m, 2H; Ar‐H, H‐4 and H‐3’’;), 7.62 (dd, ^*4*^ *J* = 2.0 Hz; 1H, Ar‐H, H‐7), 7.73 (dd, ^*3*^ *J* = 7.8 Hz, 1H, Ar‐H, H‐6’’;), 9.91 (br s, 1H, NH). ^13^C NMR (126 MHz, DMSO‐*d*_6_): *δ*= 21.3 (CH_3_), 36.5 (s, 1C, ‐SCH_2_CO‐), 47.3 (NCH_2_Ar), 110.4 (C‐7), 116.7 (C‐2’’;), 119.4 (C‐4), 122.6 (C‐5), 125.1 (C‐2^′^ and C‐6^′^), 125.9 (C‐6’’;), 127.0 (C‐4’’;), 127.2 (C‐6), 128.6 (C5’’;),129.8 (C‐4^′^), 133.1 (C‐3’’;), 136.1 (C‐1^′^), 136.2 (C‐1’’;), 137.6 (C‐7a), 138.3 (C‐3^′^ and C‐5^′^), 141.9 (C‐3a), 153.2 (C‐2), 166.9 (CO). Elemental analysis calcd for C_24_H_21_BrClN_3_OS: C 55.99, H 4.11, N 8.16, found: C 55.97, H 4.15, N 8.11.

### 2‐(6‐chloro‐1‐(3,5‐dimethylbenzyl)‐1H‐benzo[d]imidazol‐2‐ylthio)‐*N*‐(2‐chloro‐4‐methylphenyl)acetamide (8)


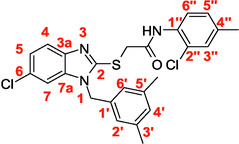


CAS Number: 1,562,665‐37‐5. M.p. 164–66 °C. ^1^H NMR (500 MHz, DMSO‐*d*_6_, ppm): *δ* = 2.17 (s, 6H, CH_3_), 2.24 (s; 3H, CH_3_), 4.32 (s, 2H; ‐SCH_2_CO‐), 5.34 (s, 2H; ‐NCH_2_Ar), 6.79 (s, 2H; Ar‐H, H‐2‘ and 6‘), 6.89 (s, 1H; Ar‐H, H‐4^′^), 7.10 (br d, ^*3*^ *J* = 8.3 Hz; 1H, Ar‐H, H‐5’’;), 7.19 (dd, ^*3*^ *J* = 8.3, ^*4*^ *J* = 2.0 Hz; 1H, Ar‐H, H‐5), 7.27 (s; 1H, H‐3’’;), 7.55 (d, ^*3*^ *J* = 8.3 Hz; 1H, Ar‐H, H‐6’’;), 7.65 (d, ^*4*^ *J* = 2.0 Hz; 1H, Ar‐H, H‐7), 7.69 (d, *J* = 8.3 Hz; 1H, Ar‐H, H‐4), 9.97 (br s, 1H, ‐NH‐). ^13^C NMR (126 MHz, DMSO‐*d*_6_): *δ* = 20.5 (CH_3_), 21.3 (2, CH_3_), 36.5 (SCH_2_CO), 47.3 (NCH_2_Ar), 110.5 (C‐7), 119.3 (C‐4), 122.6 (C‐5), 125.0 (C‐2’’;), 125.1 (C‐2^′^ and C‐6^′^), 125.5 (C‐6’’;), 127.00 (C‐6), 128.5 (C‐5’’;), 129.8 (C‐4^′^), 130.0 (C‐3’’;), 132.5 (C‐1’’;), 136.1 (C‐4’’;), 136.3(C‐1^′^), 137.6 (C‐7a), 138.3 (C‐3^′^ and C‐5^′^), 141.9 (C‐3a), 153.3 (C‐2), 166.9 (CO). Elemental analysis calcd for C_25_H_23_Cl_2_N_3_OS: C 61.98, H 4.79, N 8.67, found: C 68.76, H 4.66, N 8.50.

### 2‐(6‐Chloro‐1‐(3,5‐dimethylbenzyl)‐1H‐benzo[d]imidazol‐2‐ylthio)‐*N*‐(2‐chloro‐4‐(methylsulfonyl)phenyl)acetamide (9)


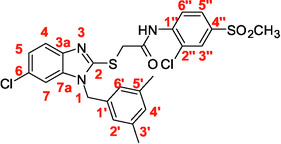


CAS Number: 1,562,665‐64‐8. M.p. 224–26 °C. ^1^H NMR (500 MHz, DMSO‐*d*_6_, ppm): *δ* = 2.17 (s, 6H, CH_3_), 3.22 (s; 3H, −SO_2_CH_3_), 4.39 (s; 2H, SCH_2_CO), 5.35 (s; 2H, NCH_2_Ph‐), 6.79 (s; 2H, Ar‐H, H‐2‘ and 6‘), 6.89 (s; 1H, ArH, H‐4^′^), 7.21 (dd, ^*3*^ *J* = 8.8 Hz, ^*4*^ *J* = 12.0 Hz; 1H, Ar‐H, H‐5), 7.56 (d, ^*3*^ *J* = 8.8 Hz; 1H, Ar‐H, H‐4), 7.62 (d, ^*4*^ *J* = 2.0 Hz; 1H, Ar‐H, H‐7), 7.84 (dd, ^*3*^ *J* = 8.3 Hz, ^*4*^ *J* = 2.0 Hz; 1H, Ar‐H, H‐5’’;), 7.98 (d, ^*4*^ *J* = 2.0 Hz; 1H, Ar‐H, H‐3’’;), 8.24 (d, ^*3*^ *J* = 8.3 Hz; 1H, Ar‐H, H‐6’’;), 10.37 (br s; 1H, NH).^13^C NMR (126 MHz, DMSO‐*d*_6_): *δ* = 21.3 (CH_3_), 21.3 (CH_3_), 36.6 (S*C*H_2_CO), 43.9 (SO_2_CH_3_), 47.4 (NCH2Ar), 110.5 (C‐7), 119.3 (C‐4), 122.7 (C‐5), 123.9 (C‐5’’;), 124.7 (C‐6’’;), 125.0 (C‐2^′^ and C‐6^′^), 125.1 (C‐2’’;), 127.1 (C‐6), 128.8 (C‐3’’;), 129.8 (C‐4^′^), 136.1 (C‐1^′^), 137.4 (C‐1’’;) 137.6 (C‐7a), 138.3 (C‐3^′^ and C‐5^′^), 139.7 (C‐4’’;), 141.8 (C‐3a), 153.2 (C‐2), 167.8 (CO). Elemental analysis calcd for C_25_H_23_Cl_2_N_3_O_3_S_2_: C 54.74, H 4.23, N 7.66, found: C 54.79, H 4.25, N 7.62.

### Relative Lipophilicity Determination

The experimental determination of relative lipophilicity was carried out using reversed‐phase thin‐layer chromatography (20 × 20 cm plates coated with C18 silica RP18 F254, E. Merck, Darmstadt, Germany). A binary solvent system consisting of acetone–water (2/1, v/v) served as the mobile phase. Compounds **1**–**9** were prepared in methanol to obtain a concentration of 2.0 mg/mL; from these working solutions, a volume of 0.2 μL was spotted on the plate, which was dried at 105 °C for 1 h before use. The chromatograms were developed to a distance of 10 cm from the origin in ascending TLC chambers and developed at 25 °C. After development, compound spots were visualized under UV light at 254 nm. Each determination was performed as triplicate 3 by spotting the compounds on the plate in distinct position; therefore, each RF value resulted as a mean value obtained in triplicate. The retardation coefficients (RF) were visualized from the chromatograms, and the relative lipophilicity RM values were calculated from the experimental RF values according to the formula RM = log[(1/ RF − 1], as shown in **Table** **2**; the higher RM values indicated higher lipophilicity. Oleanolic acid was employed as a reference in the biological essay; its logP value was in silico assessed, yielding a value of 7.20.

**Table 2:** RM‐based lipophilicity data for compounds **1–9**.

Cmpd	RM	cLoGPa)	Cmpd	RM	cLoGPa)	Cmpd	RM	cLoGPa)
**1**	1.023	5.71	**4**	0.678	4.73	**7**	1.195	6.41
**2**	0.971	5.47	**5**	0.558	4.59	**8**	1.121	6.68
**3**	0.938	4.97	**6**	1.221	6.20	**9**	1.023	5.21

^a)^ cLogP calculated by ACDLab (version 2024.2.3 Accession date July 9, 2025).

### Elastase Inhibition Assay

Elastase inhibition was determined using the substrate *N*‐succ‐(Ala)3‐nitroanilide (SANA) as previously described.^[^ ^27^ ^]^ The assay was performed in 0.1 M Tris‐HCl buffer (pH 8.0). PPE (3.3 µg mL^−1^) was incubated with or without the compound for 20 min, and after incubation, the substrate (1.6 mM) was added. The release of *p*‐nitroaniline during cleavage of the substrate SANA by the enzyme activity was monitored at 410 nm. The control was performed with DMSO, while oleanolic acid was used as a positive control. The IC_50_ value, a concentration giving 50% inhibition of elastase activity, was determined by interpolation of dose–response curves. The mode of inhibition on the enzyme was performed using the Lineweaver–Burk plot. Different concentrations of SANA (0.5, 0.75, 1.0, and 1.5 mM) were used for the assay. All reagents were purchased from “Merck Sigma Aldrich” (Milano, Italy).

### In Silico Studies

Molecular docking studies were conducted following a previously validated protocol.^[^ ^22^ ^]^ For clarity, a consistent nomenclature for the binding site must be established. In the renumbered elastase structure, amino acid residues were assigned sequentially from Val 16 (16) to Asn255 (245), with the catalytic triad defined as Ser203 (195), His60 (57), and Asp108 (102), in accordance with previous work.^[^ ^22^ ^]^ Flexible docking simulations were carried out with software GOLD v2024.^[^ ^28^ ^]^ The following amino acidic residues were defined as flexible: His60, Val103, Gln200, Ser203, Ser222, Phe223, and Val224. The docking grid box was centered at the coordinates x: −12.2628, y: 20.1692, z: 37.7359 with a radius of 10 Å. The "Allow early termination" option was disabled, and each ligand was subjected to 100 runs. The CHEMPLP scoring function was applied. The protein structure was prepared using the Protein Preparation Wizard (Schrodinger 2025–3) in the Schrödinger suite,^[^ ^29^ ^]^ applying the default settings. Missing side chains and loops were added using the Prime module.^[^ ^30^ ^]^

Ligand structures were prepared using the LigPrep tool of the Schrödinger suite (Schrodinger 2025–3), with ionization states calculated at pH 7.0 ± 2.0 using Epik.^[^ ^37^ ^]^ The best‐ranked docking pose for each compound was selected and further analyzed for protein–ligand interactions using Maestro (Schrodinger 2025–3)

Binding free energy calculations were performed using the MM‐GBSA approach, as implemented in the Prime module of the Schrödinger suite.^[^ ^30^ ^]^ The Variable Surface Generalized Born implicit solvent model was employed in combination with the OPLS2005 force field. Calculations were carried out on protein–ligand complexes obtained from flexible molecular docking. All complexes were previously minimized using the Refine Protein–Ligand Complex utility available in Schrödinger suite (Schrodinger 2025–3). This minimization step ensures the attenuation of local steric clashes and optimizes the conformations of side chains and ligands at the binding site.

Molecular dynamics simulations were carried out in triplicate using Desmond tool^[^ ^29^ ^]^ in the Schrödinger suite. Each analysis was performed using an orthorhombic simulation box (10 Å × 10 Å × 10 Å) and TIP3P water model. Counterions (Na^+^ and Cl^−^) were added to neutralize the total system charge, maintaining a physiological salt concentration of 0.15 M. OPLS5 was used as force field. MD simulations were carried out using the NPT (constant number of particles, pressure, and temperature) ensemble, maintaining constant temperature (300 K) and pressure (1 atm). Each simulation was run for 150 500 ns and the resulting trajectories were analyzed using the Simulation Interaction Diagram tool (Schrodinger 2025–3). In silico prediction of skin permeability, skin sensitization, and mutagenicity (Ames test) was performed for the most promising compounds (**6** and **7**) using pkCSM.^[^ ^31^ ^]^ In parallel, SwissADME^[^ ^32^ ^]^ was used to assess PAINS alerts and Brenk structural alerts. These filters are commonly used to evaluate lead‐likeness and guide compound optimization.

## Supporting Information

The data that support the findings of this study are available in the supplementary material of this article.

## Conflict of Interest

The authors declare no conflict of interest.

## Author Contributions

**Francesca Mancuso** and **Giulia Savoca** carried out synthetic procedure; **Sonia Floris** and **Antonella Fais** conducted enzymatic assays on elastase; **Giovanna Pitasi** performed the computational analysis; **Rosaria Gitto**, **Antonella Fais**, and **Laura De Luca** wrote the manuscript with input from all authors; and all authors reviewed and approved the final version of the manuscript.

## Supporting information

Supplementary Material

## Data Availability

The data that support the findings of this study are available in the supplementary material of this article.

## References

[1] R. Tenchov , J. M. Sasso , X. Wang , Q. A. Zhou , ACS Chem. Neurosci. 2024, 15, 1. 38095562 10.1021/acschemneuro.3c00531 PMC10767750

[2] Z. Werb , M. J. Banda , J. H. McKerrow , R. A. Sandhaus , J. Invest. Dermatol. 1982, 79, 154s. 7045242 10.1111/1523-1747.ep12546056

[3] M. Rai , M. Curley , Z. Coleman , F. Demontis , Aging Cell 2022, 21, e13603. 35349763 10.1111/acel.13603 PMC9124314

[4] J. S. Ocampo‐Gallego , D. Pedroza‐Escobar , A. R. Caicedo‐Ortega , M. T. Berumen‐Murra , A. L. Novelo‐Aguirre , R. D. de Sotelo‐León , D. Delgadillo‐Guzmán , Fundam. Clin. Pharmacol. 2024, 38, 13. 37609718 10.1111/fcp.12946

[5] S. Ahmad , M. Saleem , N. Riaz , Y. S. Lee , R. Diri , A. Noor , D. Almasri , A. Bagalagel , M. F. Elsebai , Front. Pharmacol. 2020, 11, 688. 32581778 10.3389/fphar.2020.00688 PMC7291377

[6] L. Shaw , O. Wiedow , Biochem. Soc. Trans. 2011, 39, 1450. 21936832 10.1042/BST0391450

[7] H. Ohbayashi , Expert Opin. Investig. Drugs. 2002, 11, 965. 10.1517/13543784.11.7.965 12084007

[8] K. Ohmoto , T. Yamamoto , M. Okuma , T. Horiuchi , H. Imanishi , Y. Odagaki , K. Kawabata , T. Sekioka , Y. Hirota , S. Matsuoka , H. Nakai , M. Toda , J. C. Cheronis , L. W. Spruce , A. Gyorkos , M. Wieczorek , J. Med. Chem. 2001, 44, 1268. 11312926 10.1021/jm000410y

[9] M. G. Matera , P. Rogliani , J. Ora , L. Calzetta , M. Cazzola , Expert Opin. Investig. Drugs 2023, 32, 793. 10.1080/13543784.2023.2263366 37740909

[10] N. Tsuji , S. Moriwaki , Y. Suzuki , Y. Takema , G. Imokawa , Photochem. Photobiol. 2001, 74, 283. 11547567 10.1562/0031-8655(2001)074<0283:troesb>2.0.co;2

[11] W. Bode , E. Meyer , J. C. Powers , Biochemistry 1989, 28, 1951. 2655701 10.1021/bi00431a001

[12] M. Würtele , M. Hahn , K. Hilpert , W. Höhne , Acta Crystallogr. D Biol. Crystallogr. 2000, 56, 520. 10739939 10.1107/s0907444900000299

[13] M. A. Navia , B. M. McKeever , J. P. Springer , T. Y. Lin , H. R. Williams , E. M. Fluder , C. P. Dorn , K. Hoogsteen , Proc. Natl. Acad. Sci. U S A. 1989, 86, 7. 2911584 10.1073/pnas.86.1.7 PMC286392

[14] M. A. A. Shakila , R. A. Ur , S. Z. Siddiqui , M. Nazir , S. Muhammad , H. Raza , S. A. A. Shah , M. Shahid , A. R. Chaudhry , S. J. Kim , Chem. Biodivers. 2025, 22, e202401806. 39572384 10.1002/cbdv.202401806

[15] I. Kurt‐Celep , S. Ahmed , M. V. Cetiz , A. I. Uba , G. Ak , S. Selvi , A. Emir , S. Dall’Acqua , G. Zengin , Arch. Pharm. 358, 2025, e70057. 10.1002/ardp.70057 40726230

[16] M. I. Tousif , M. Nazir , N. Riaz , M. Saleem , S. Tauseef , S. M. Azam , M. A. Yawer , G. Zengin , Chembiochem 2023, 24, e202300346. 37642535 10.1002/cbic.202300346

[17] K. Jakimiuk , J. Gesek , A. G. Atanasov , M. Tomczyk , J. Enzyme. Inhib. Med. Chem. 2021, 36, 1016. 33980119 10.1080/14756366.2021.1927006 PMC8128182

[18] L. Marinaccio , A. Stefanucci , G. Scioli , A. D. Valle , G. Zengin , A. Cichelli , A. Mollica , Int. J. Mol. Sci. 2022, 23. 35328340 10.3390/ijms23062924 PMC8954713

[19] C. D. Papaemmanouil , J. Peña‐García , A. J. Banegas‐Luna , A. D. Kostagianni , I. P. Gerothanassis , H. Pérez‐Sánchez , A. G. Tzakos , Antioxidants 2022, 11, 2268. 36421454 10.3390/antiox11112268 PMC9686885

[20] P. Ledwon , A. M. Papini , P. Rovero , R. Latajka , Materials 2021, 14, 3217. 34200889 10.3390/ma14123217 PMC8230458

[21] L. Crocetti , M. T. Quinn , I. A. Schepetkin , M. P. Giovannoni , Expert Opin. Ther. Pat. 2019, 29, 555. 31204543 10.1080/13543776.2019.1630379 PMC9642779

[22] G. Pitasi , A. Brancale , S. Floris , A. Fais , R. Gitto , L. De Luca , Int. J. Mol. Sci. 2024, 25, 11174. 39456954 10.3390/ijms252011174 PMC11508974

[23] A. M. Monforte , S. Ferro , L. De Luca , G. L. Surdo , F. Morreale , C. Pannecouque , J. Balzarini , A. Chimirri , Bioorg. Med. Chem. 2014, 22, 1459. 24457088 10.1016/j.bmc.2013.12.045

[24] G. Mandalari , C. Bisignano , A. Smeriglio , M. Denaro , M. Musarra‐Pizzo , R. Pennisi , F. Mancuso , S. Ferro , D. Trombetta , A. M. Monforte , M. T. Sciortino , L. De Luca , PLoS One 2019, 14, e0216384. 31048874 10.1371/journal.pone.0216384 PMC6497310

[25] D. N. Kommi , D. Kumar , R. Bansal , R. Chebolu , A. K. Chakraborti , Green Chem. 2012, 14, 3329.

[26] F. Mancuso , A. Di Fiore , L. De Luca , A. Angeli , S. M. Monti , G. De Simone , C. T. Supuran , R. Gitto , ACS Med. Chem. Lett. 2020, 11, 1000. 32435417 10.1021/acsmedchemlett.0c00062 PMC7236538

[27] B. Era , S. Floris , V. Sogos , C. Porcedda , A. Piras , R. Medda , A. Fais , F. Pintus , Plants 2021, 10, 151. 33466576 10.3390/plants10010151 PMC7828731

[28] G. Jones , P. Willett , R. C. Glen , A. R. Leach , R. Taylor , J. Mol. Biol. 1997, 267, 727. 9126849 10.1006/jmbi.1996.0897

[29] K. J. Bowers , E. Chow , H. Xu , R. O. Dror , M. P. Eastwood , B. A. Gregersen , J. L. Klepeis , I. Kolossvary , M. A. Moraes , F. D. Sacerdoti , J. K. Salmon , Y. Shan , D. E. Shaw , Proc. of the 2006 ACM/IEEE Conf. on Supercomputing, Association for Computing Machinery, Tampa, Florida 2006.

[30] M. P. Jacobson , D. L. Pincus , C. S. Rapp , T. J. Day , B. Honig , D. E. Shaw , R. A. Friesner , Proteins 2004, 55, 351. 15048827 10.1002/prot.10613

[31] D. E. Pires , T. L. Blundell , D. B. Ascher , J. Med. Chem. 2015, 58, 4066. 25860834 10.1021/acs.jmedchem.5b00104 PMC4434528

[32] A. Daina , O. Michielin , V. Zoete , Sci. Rep. 2017, 7, 42717. 28256516 10.1038/srep42717 PMC5335600

[33] M. L. Barreca , A. Rao , L. De Luca , N. Iraci , A. M. Monforte , G. Maga , E. De Clercq , C. Pannecouque , J. Balzarini , A. Chimirri , Bioorg. Med. Chem. Lett. 2007, 17, 1956. 17276064 10.1016/j.bmcl.2007.01.025

[34] H. J. Yoon , S. J. Yang , Y. D. Gong , ACS Comb. Sci. 2017, 19, 738. 29095593 10.1021/acscombsci.7b00106

[35] A. M. Monforte , A. Rao , P. Logoteta , S. Ferro , L. De Luca , M. L. Barreca , N. Iraci , G. Maga , E. De Clercq , C. Pannecouque , A. Chimirri , Bioorg. Med. Chem. 2008, 16, 7429. 18585918 10.1016/j.bmc.2008.06.012

[36] K. Oh , H. Kim , F. Cardelli , T. Bwititi , A. M. Martynow , J. Org. Chem. 2008, 73, 2432. 18278938 10.1021/jo702457t

[37] R. C. Johnston , K. Yao , Z. Kaplan , M. Chelliah , K. Leswing , S. Seekins , S. Watts , D. Calkins , J. C. Elk , S. Jerome , M. P. Repasky , J. C. Shelley , J. Chem. Theory. Comput. 2023, 19, 2380. 37023332 10.1021/acs.jctc.3c00044

