# Supplementary material for: Investigating the Ligand‐Binding Properties of N‐arylbenzimidazoles as Novel Elastase Inhibitors
Source: ChemMedChem. 2025 Dec 21;21(4):e202500879. doi: 10.1002/cmdc.202500879 (PMC12913230; doi:10.1002/cmdc.202500879)
Supplement: Supplementary file 1 — Supplementary Material [file CMDC-21-e202500879-s001.pdf]

SUPPORTING INFORMATION

Investigating the ligand-binding properties of *N*-arylbenzimidazoles as novel elastase inhibitors

Giovanna Pitasi,[a] Sonia Floris,[b] Francesca Mancuso,[a] Giulia Savoca,[a] Rosaria Gitto,[a] Antonella Fais,[b] and Laura De Luca*[a]

[a] G. Pitasi, Dr. F. Mancuso, Dr. G. Savoca, Prof. R. Gitto, Prof. L. De Luca
Department of Chemical, Biological, Pharmaceutical and Environmental Sciences
University of Messina
Viale F. Stagno D'Alcontres 31, Messina I-98125, Italy
E-mail: laura.deluca@unime.it

[b] Dr. S. Floris, Prof. A. Fais
Department of Life and Environment Sciences,
University of Cagliari
Monserrato 09042, Cagliari, Italy

Table of contents:

Figure S1-S20: 1H-NMR and 13C-NMR spectra of synthetized compounds **2-9**

Figure S21: Comparative molecular binding poses of ligands **2**–**9** and the reference compound **1** within the active site of PPE (PDB ID: 1ELE).

Figure S22: Root-mean-square deviation (RMSD) profiles of PPE–ligand complexes with compounds **6** and **7** (three 500 ns MD replicas).

Table S1: MM-GBSA results for all eight ligands (**2-9**) and reference compound **1**.

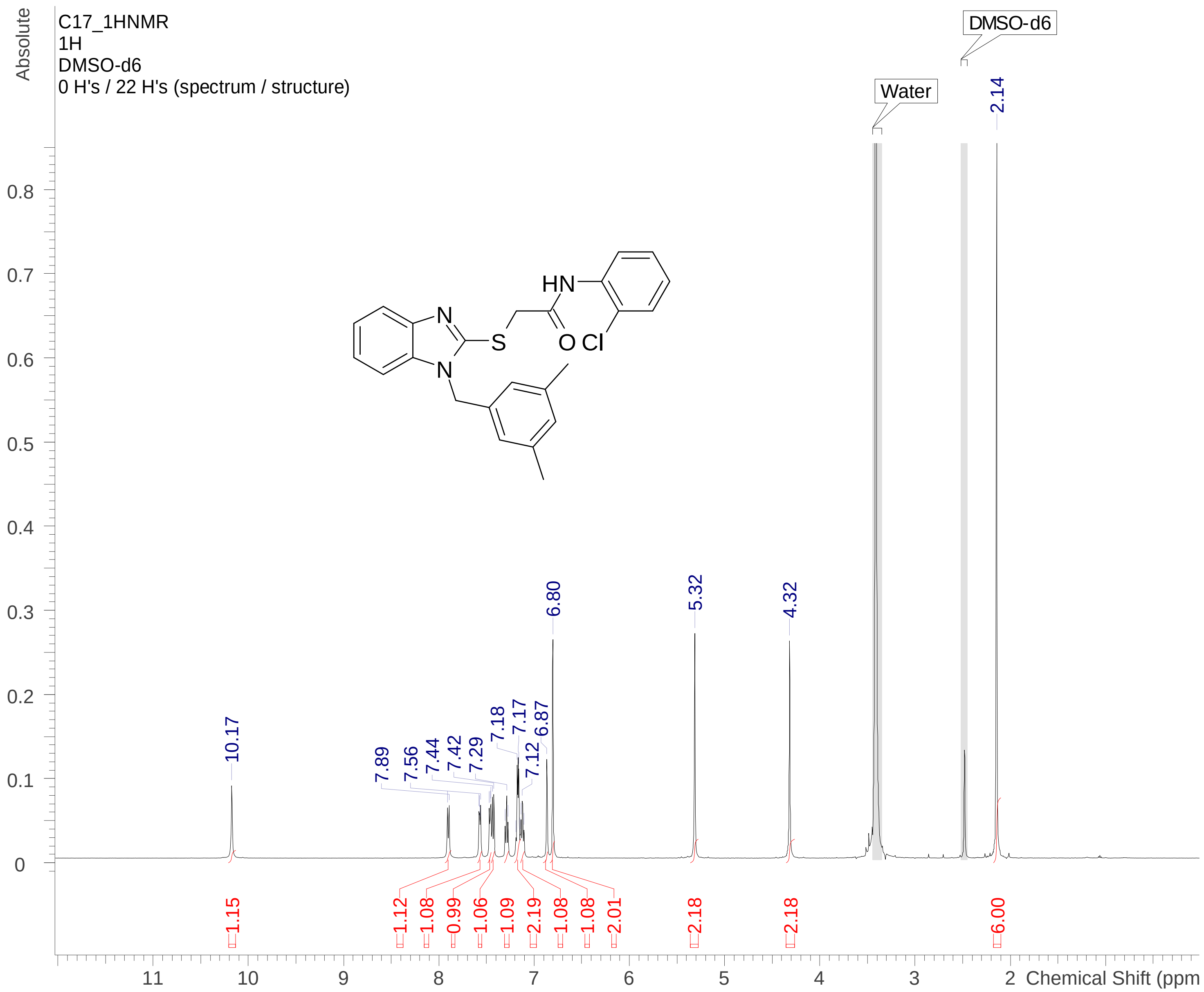

Figure S1: ^{1}H-NMR (DMSO-d_6) spectrum for *N*-(2-chlorophenyl)-2-(1-(3,5-dimethylbenzyl)-1*H*-benzo[*d*]imidazol-2-ylthio)acetamide (**2**)

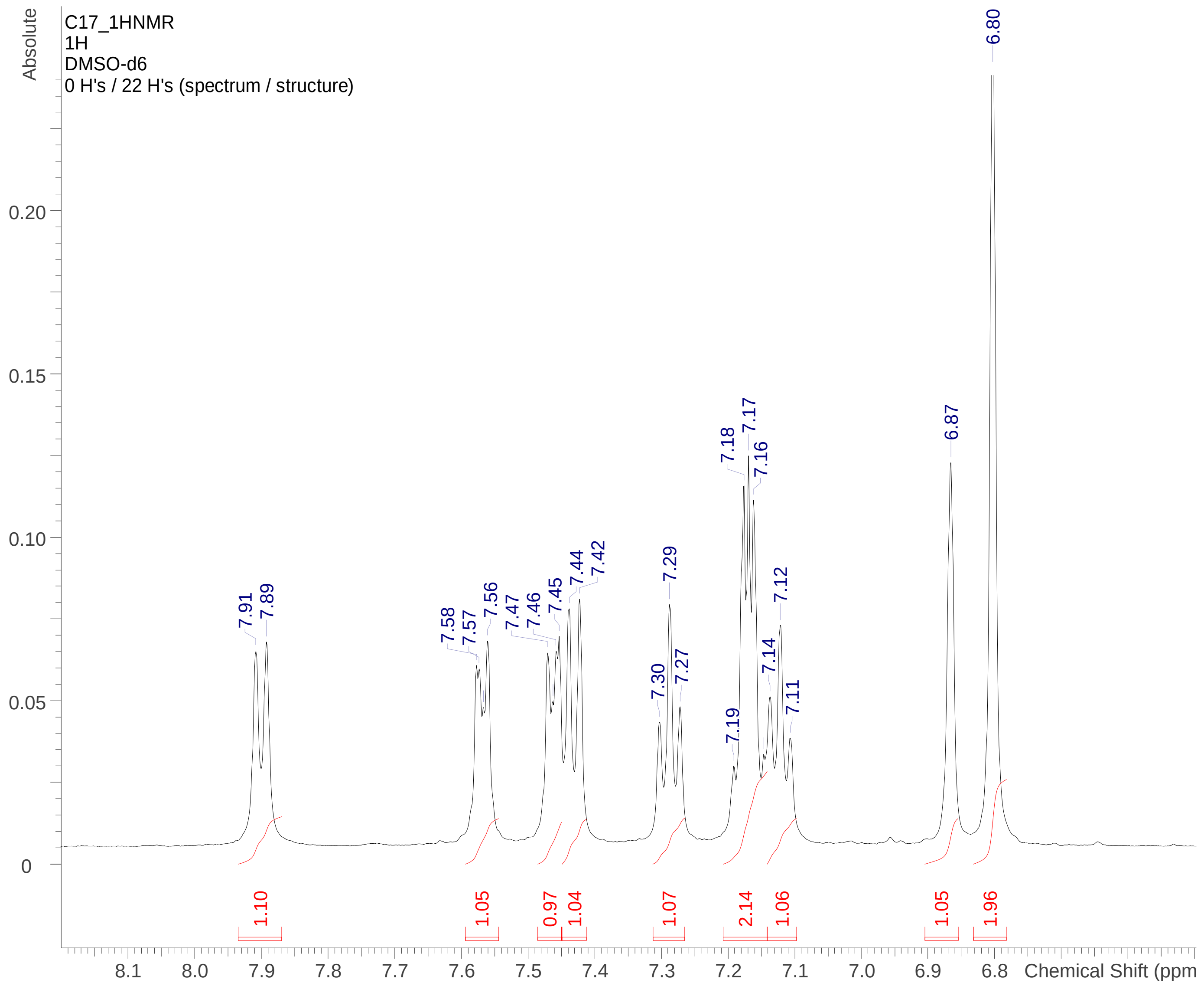

Figure S2: Expanded region of the ^{1}H NMR (DMSO-d_6) spectrum for *N*-(2-chlorophenyl)-2-(1-(3,5-dimethylbenzyl)-1*H*-benzo[*d*]imidazol-2-ylthio)acetamide (**2**) highlighting the aromatic proton signals.

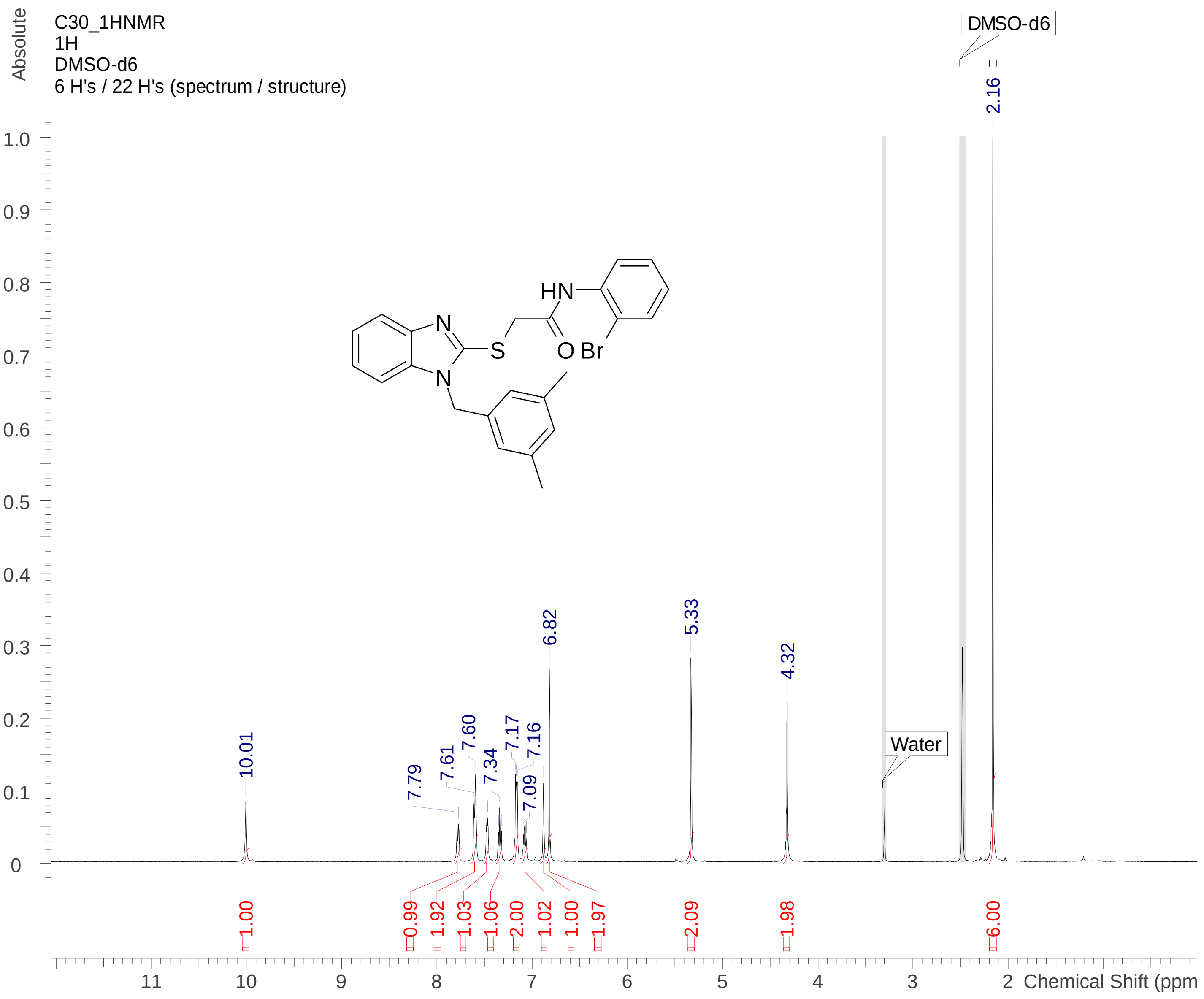

Figure S3: ^{1}H-NMR (DMSO-d_6) spectrum for *N*-(2-bromophenyl)-2-(1-(3,5-dimethylbenzyl)-1*H*-benzo[*d*]imidazol-2-ylthio)acetamide (**3**)

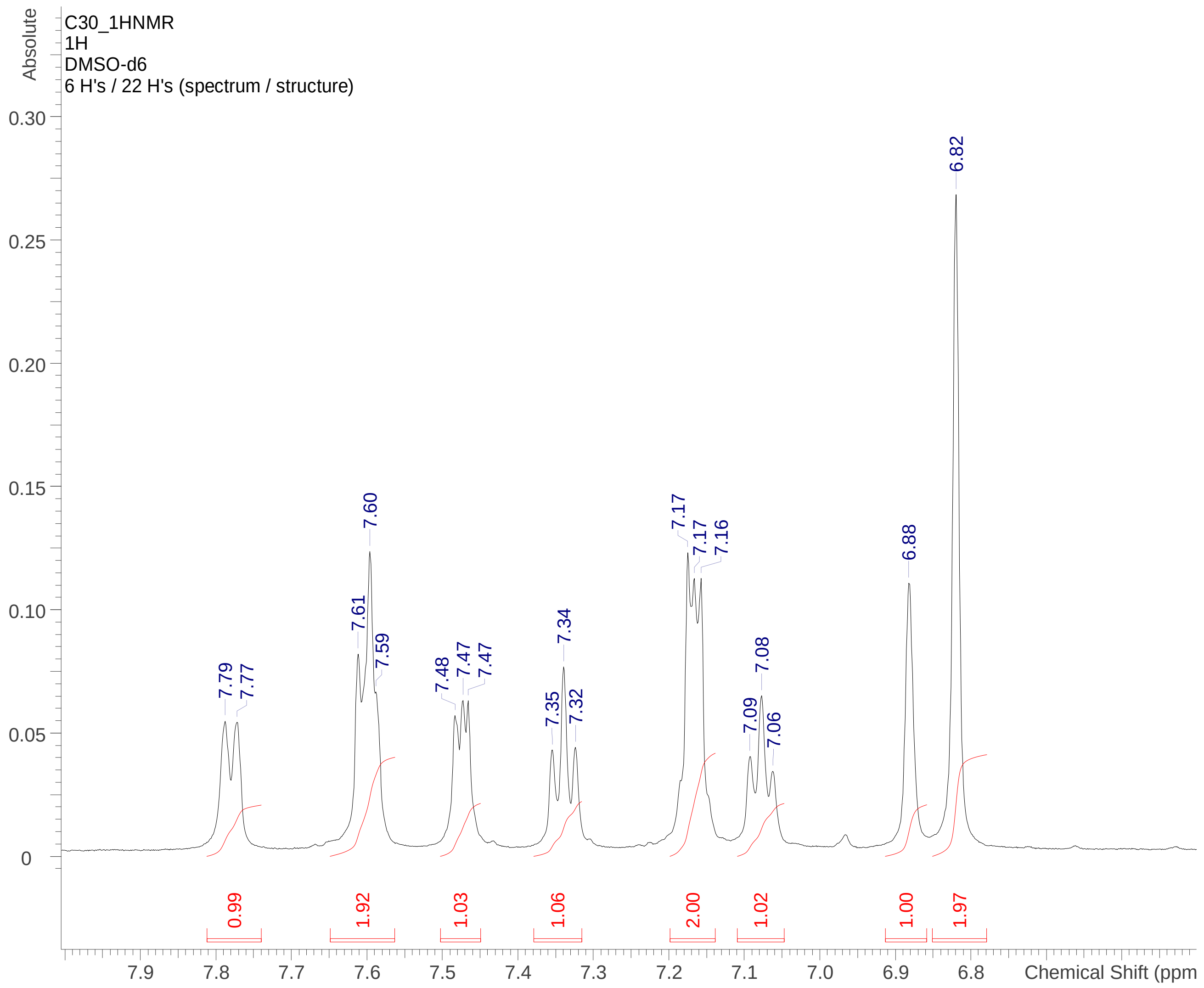

Figure S4: Expanded region of the ^{1}H NMR (DMSO-d_6) spectrum for *N*-(2-bromophenyl)-2-(1-(3,5-dimethylbenzyl)-1*H*-benzo[*d*]imidazol-2-ylthio)acetamide (**3**) highlighting the aromatic proton signals.

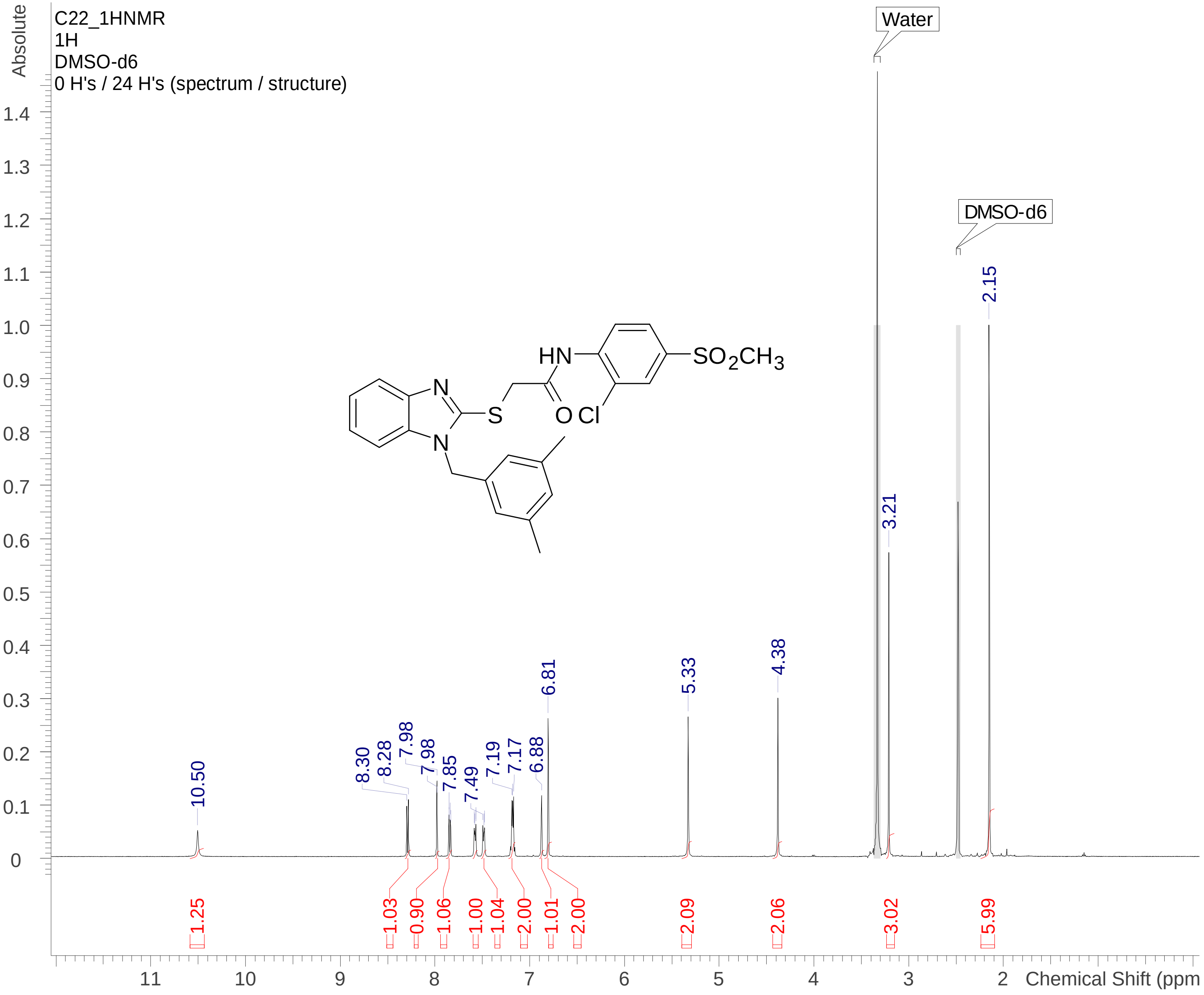
C22_1HNMR
1H
DMSO-d6
0 H's / 24 H's (spectrum / structure)
Absolute
Water
DMSO-d6
HN
SO2CH3
N
S
N
O Cl
10.50
8.30
8.28
7.98
7.98
7.85
7.49
7.19
7.17
6.88
6.81
5.33
4.38
3.21
2.15
1.25
1.03
0.90
1.06
1.00
1.04
2.00
1.01
2.00
2.09
2.06
3.02
5.99
Chemical Shift (ppm

Figure S5: ^{1}H-NMR (DMSO-d_6) spectrum for *N*-(2-chloro-4-(methylsulfonyl)phenyl)-2-(1-(3,5-dimethylbenzyl)-1H-benzo[d]imidazol-2-ylthio)acetamide (**4**)

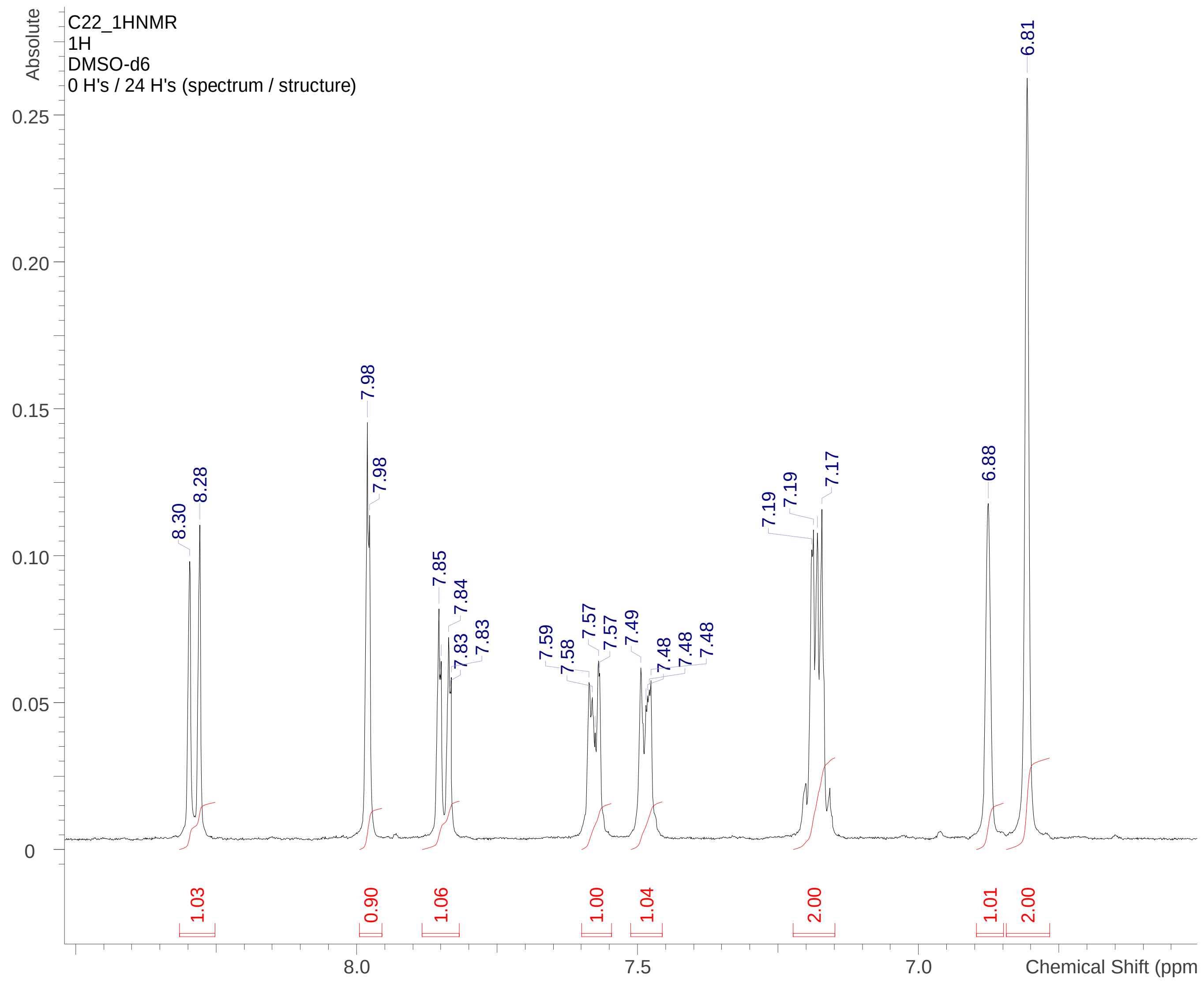

Figure S6: Expanded region of the ^{1}H NMR (DMSO-d_6) spectrum for *N*-(2-chloro-4-(methylsulfonyl)phenyl)-2-(1-(3,5-dimethylbenzyl)-1H-benzo[d]imidazol-2-ylthio)acetamide (**4**) highlighting the aromatic proton signals.

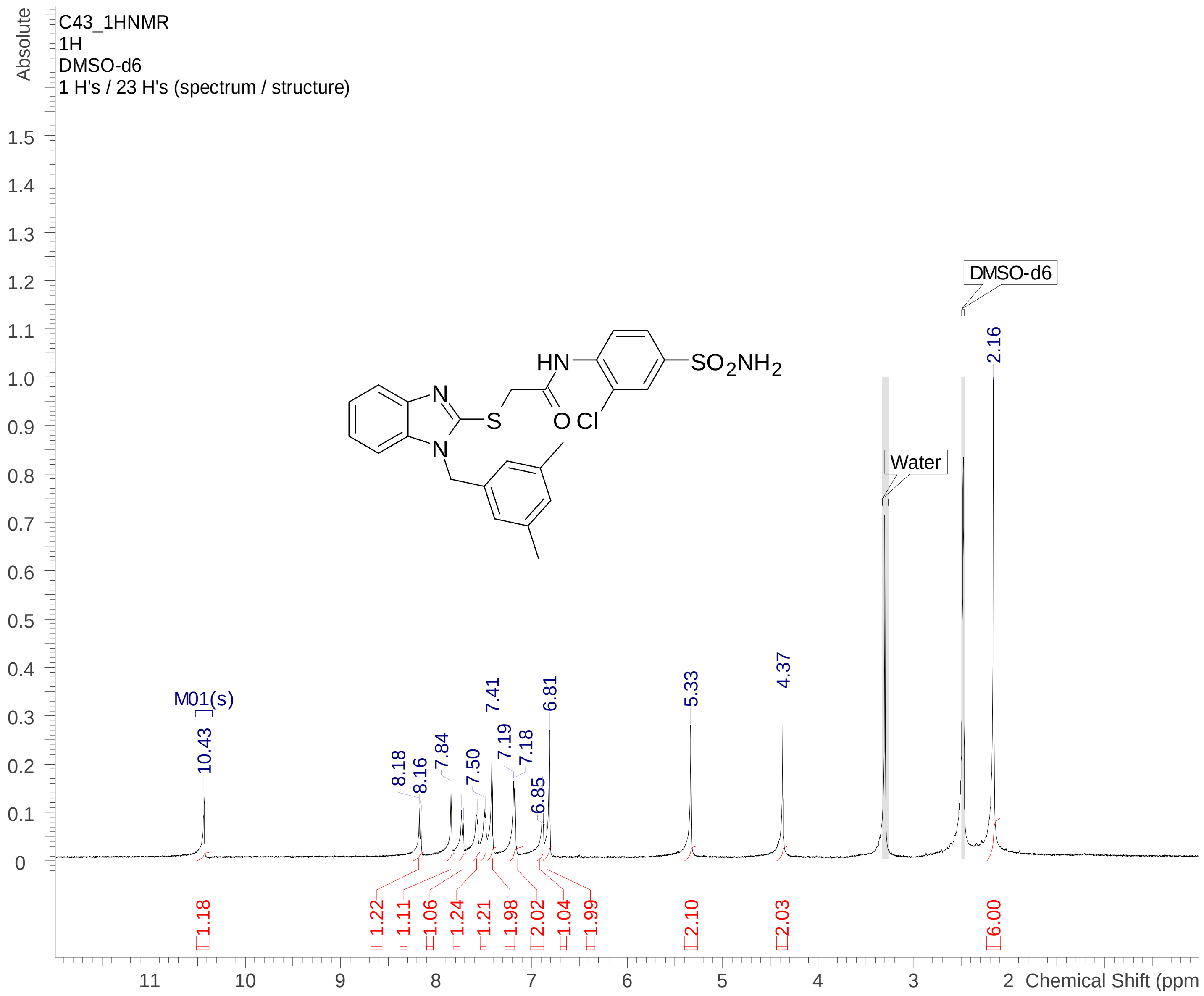

Figure S7: ^{1}H-NMR (DMSO-d_6) spectrum for N-(2-chloro-4-sulfamoylphenyl)-2-(1-(3,5-dimethylbenzyl)-1-benzo[d]imidazol-2-ylthio)acetamide (**5**)

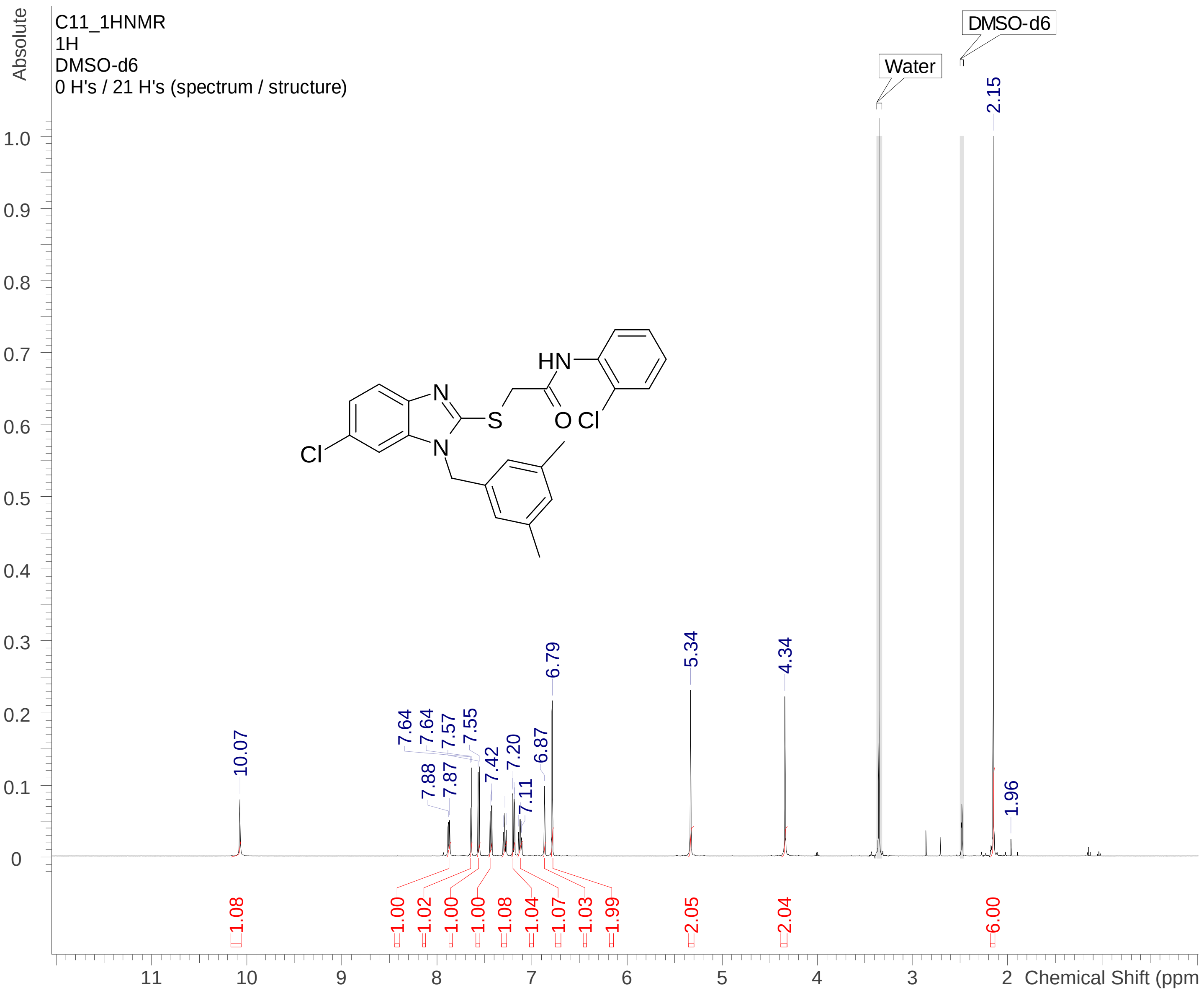

Figure S8: ^{1}H-NMR (DMSO-d_6) spectrum for 2-(6-chloro-1-(3,5-dimethylbenzyl)-1*H*-benzo[*d*]imidazol-2-ylthio)-*N*-(2-chlorophenyl)acetamide (**6**)

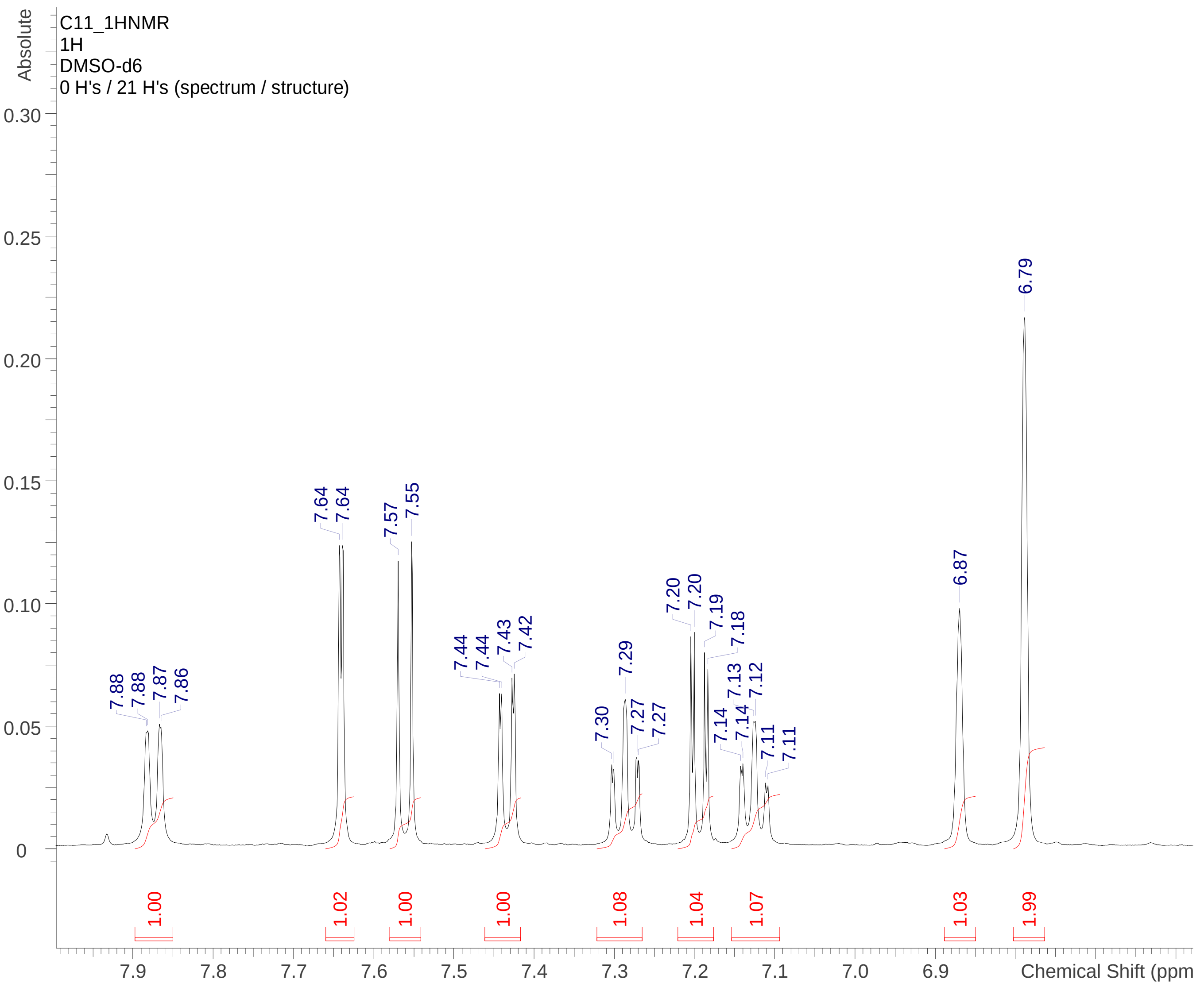

Figure S9: Expanded region of the ^{1}H NMR (DMSO-d_6) spectrum for 2-(6-chloro-1-(3,5-dimethylbenzyl)-1*H*-benzo[*d*]imidazol-2-ylthio)-*N*-(2-chlorophenyl)acetamide (6) highlighting the aromatic proton signals.

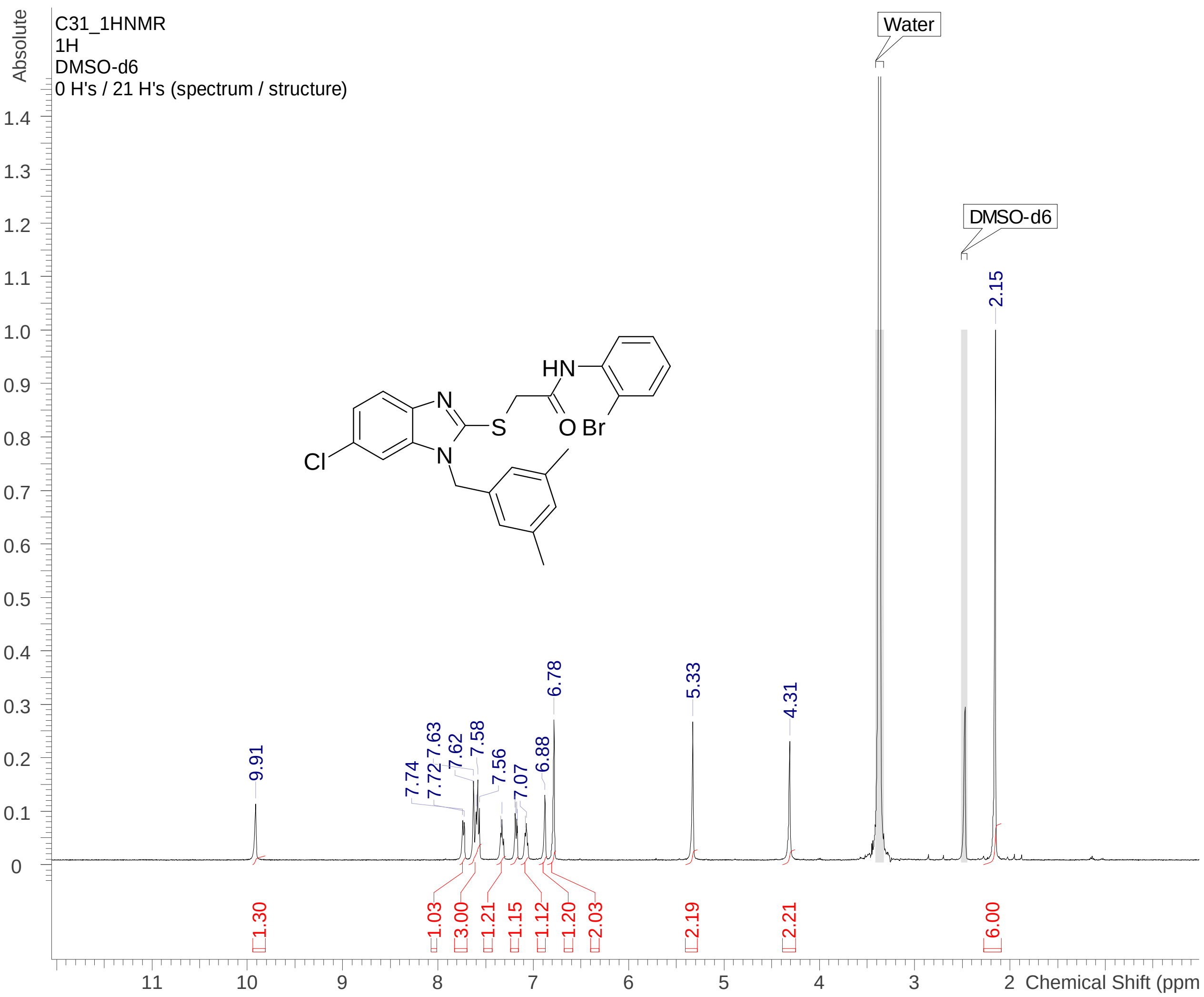

Figure S10: ^{1}H-NMR (DMSO-d_6) spectrum for *N*-(2-bromophenyl)-2-(6-chloro-1-(3,5-dimethylbenzyl)-1*H*-benzo[*d*]imidazol-2-ylthio)acetamide (**7**)

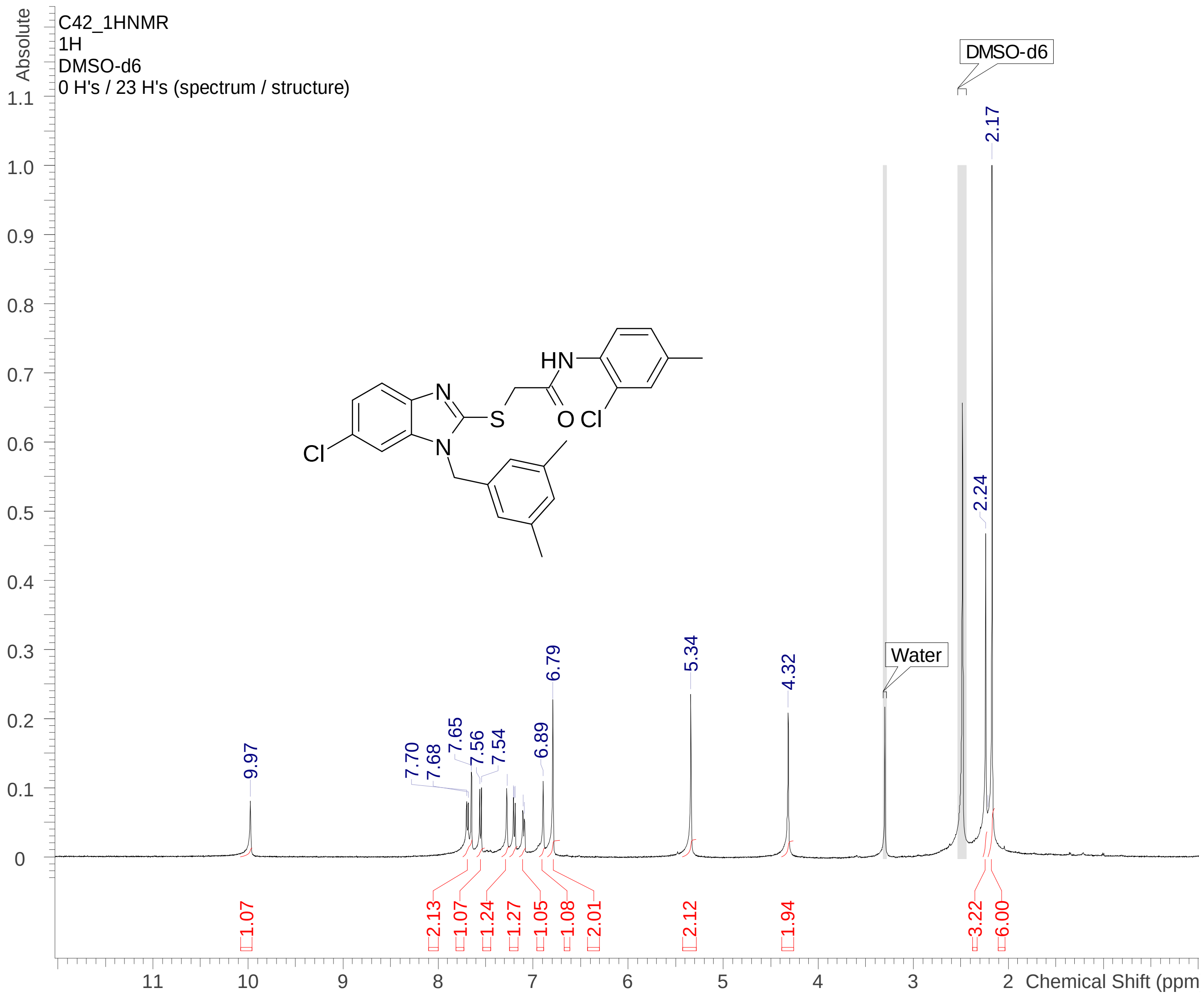

Figure S11: ¹H-NMR (DMSO-d_6) spectrum for 2-(6-chloro-1-(3,5-dimethylbenzyl)-1*H*-benzo[*d*]imidazol-2-ylthio)-*N*-(2-chloro-4-methylphenyl)acetamide (**8**)

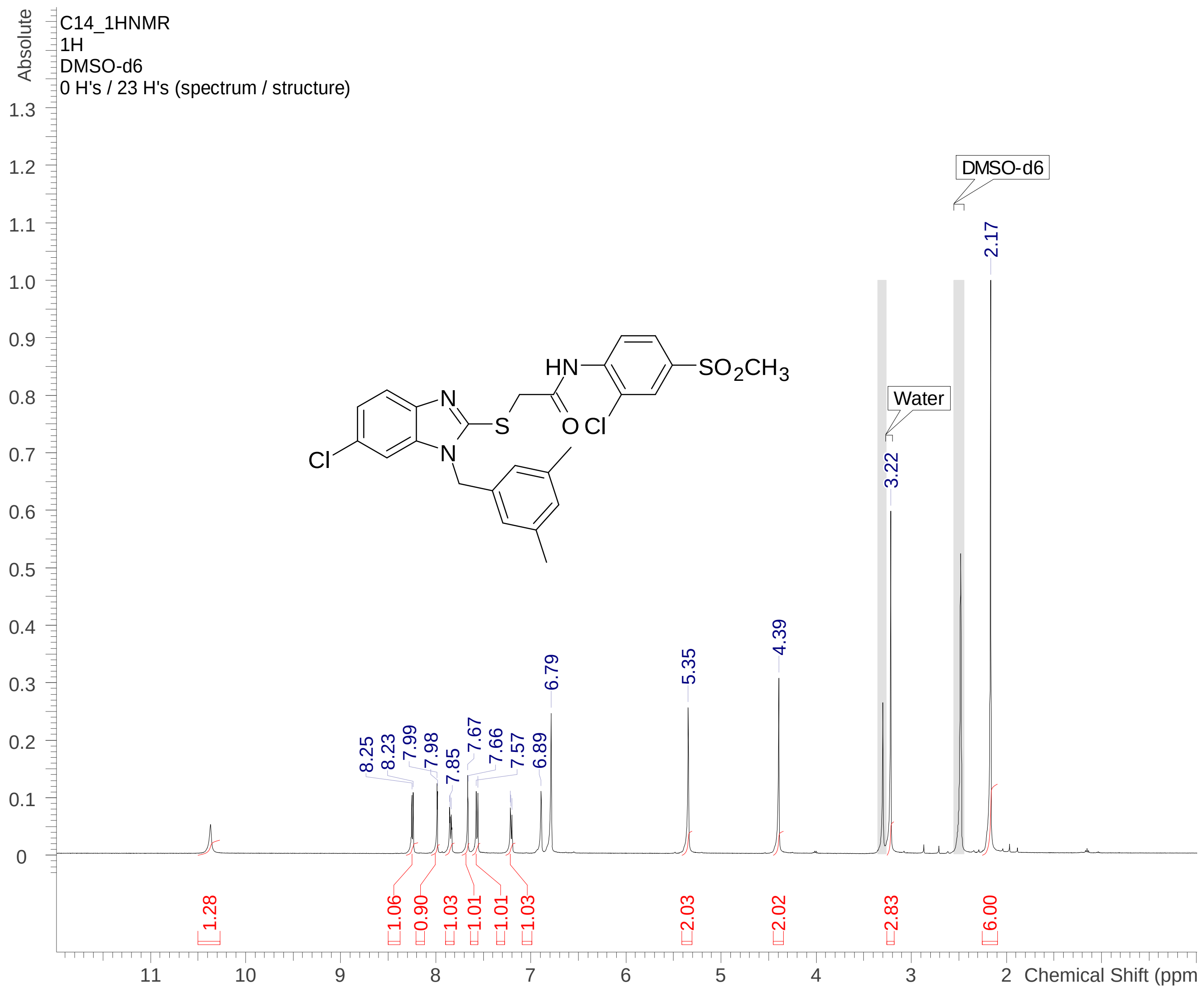

Figure S12: ^{1}H-NMR (DMSO-d_6) spectrum for 2-(6-chloro-1-(3,5-dimethylbenzyl)-1H-benzo[d]imidazol-2-ylthio)-N-(2-chloro-4-(methylsulfonyl)phenyl)acetamide (**9**)

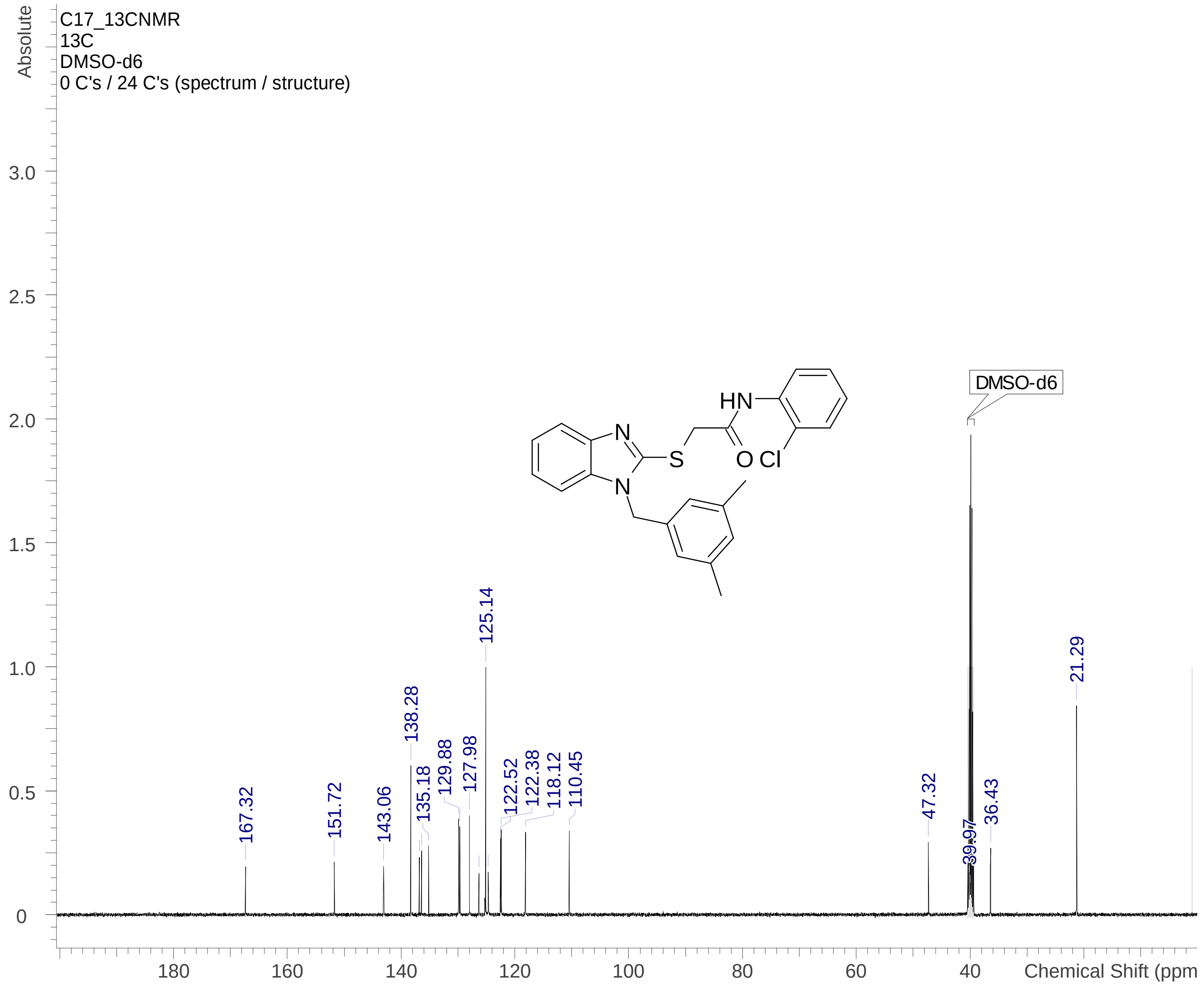

Figure S13: ^{13}C-NMR (DMSO-d_6) spectrum for *N*-(2-chlorophenyl)-2-(1-(3,5-dimethylbenzyl)-1*H*-benzo[*d*]imidazol-2-ylthio)acetamide (**2**)

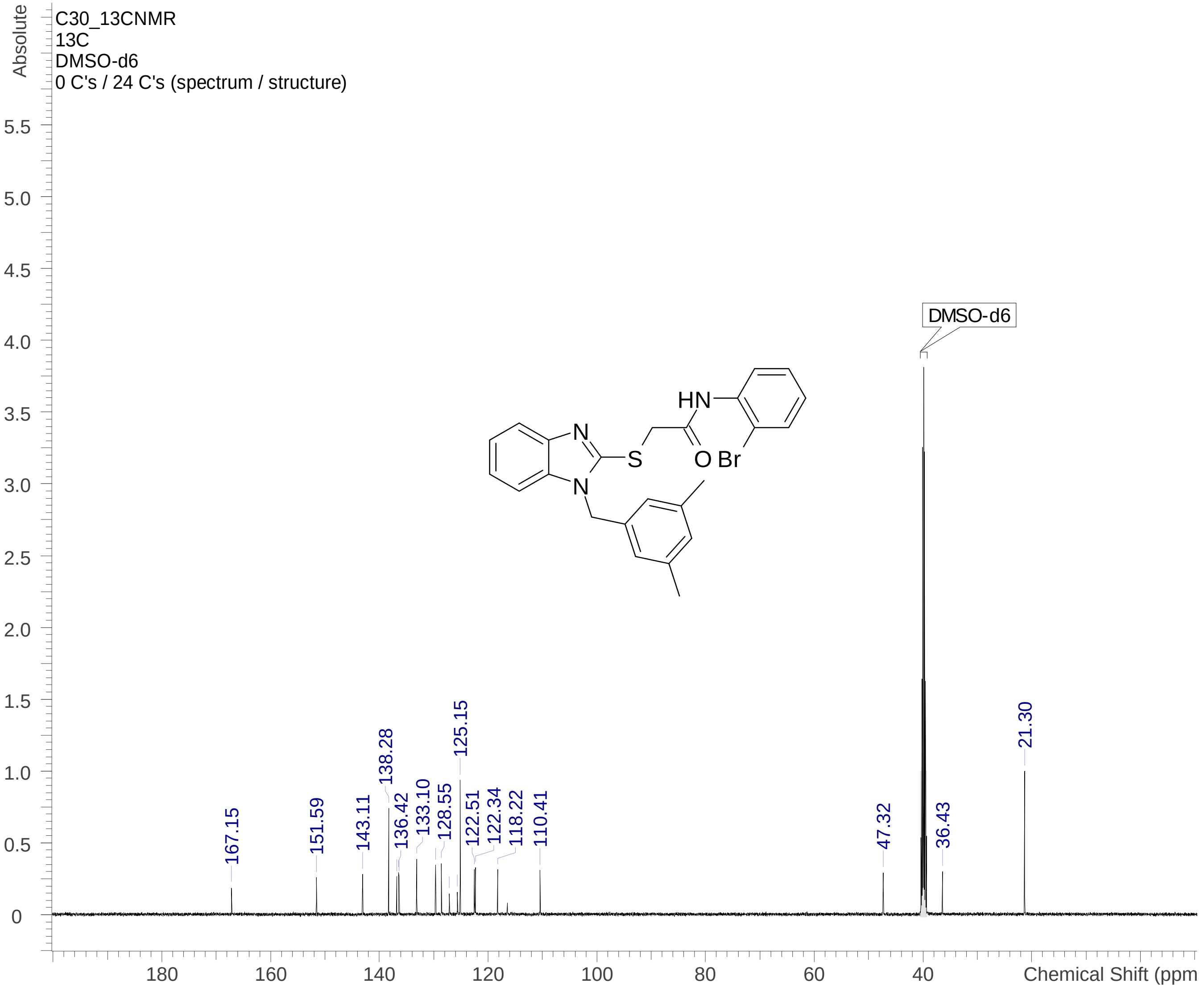

Figure S14: ^{13}C-NMR (DMSO-$d_6$spectrum for *N*-(2-bromophenyl)-2-(1-(3,5-dimethylbenzyl)-1*H*-benzo[*d*]imidazol-2-ylthio)acetamide (**3**)

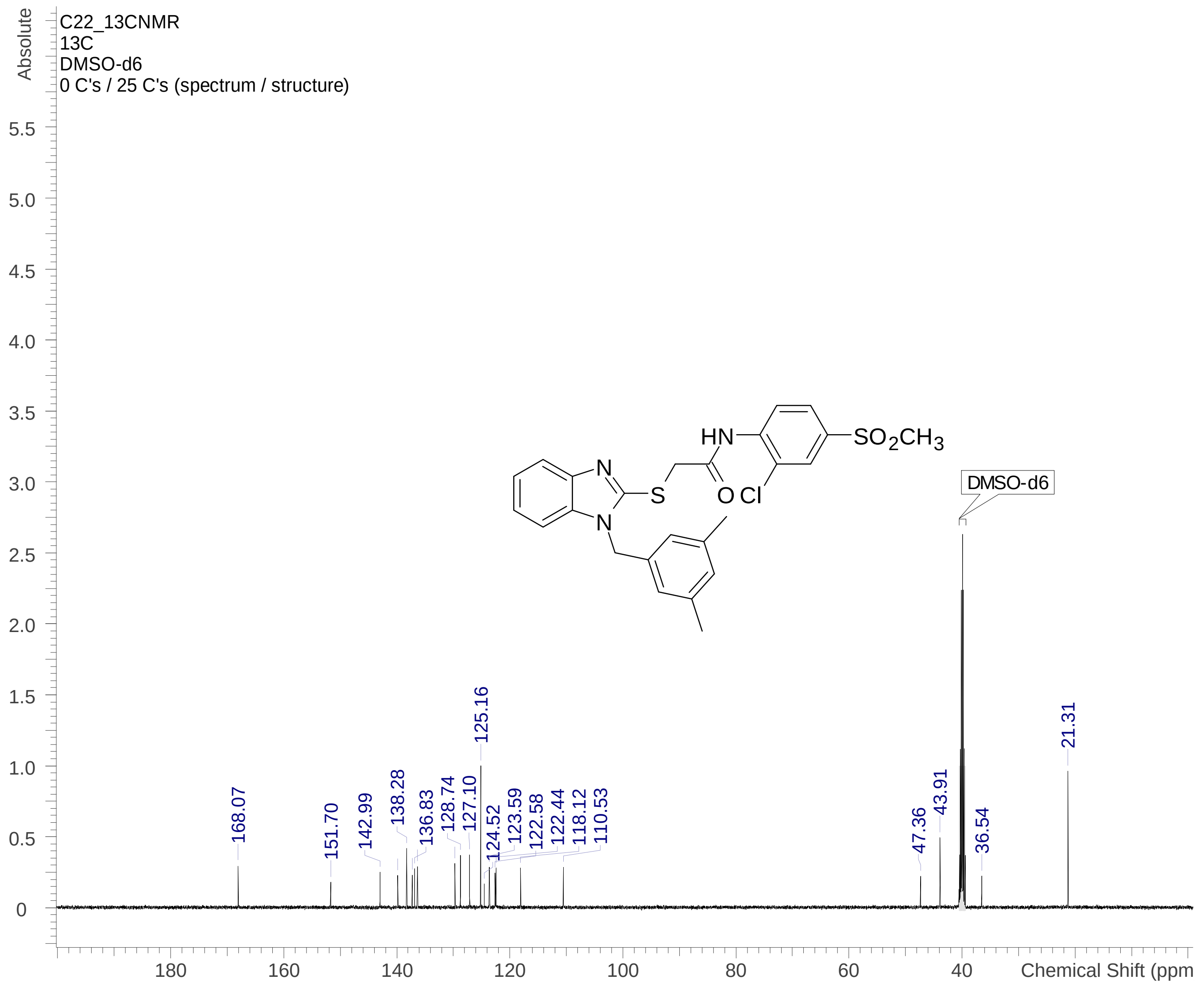

Figure S15: ^{13}C-NMR (DMSO-$d_6$spectrum for *N*-(2-chloro-4-(methylsulfonyl)phenyl)-2-(1-(3,5-dimethylbenzyl)-1H-benzo[d]imidazol-2-ylthio)acetamide (**4**)

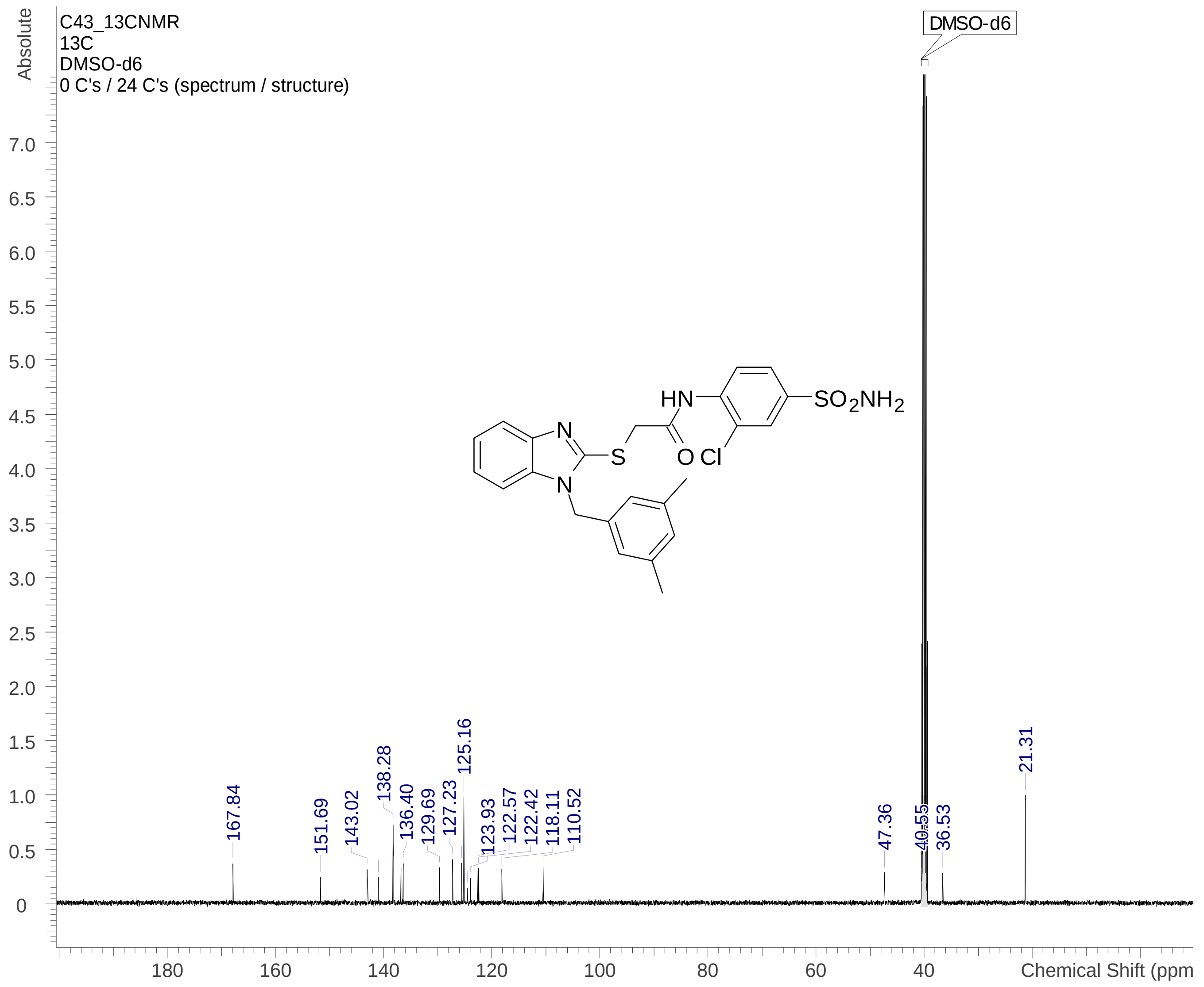

Figure S16: ^{13}C-NMR (DMSO-d_6) spectrum for N-(2-chloro-4-sulfamoylphenyl)-2-(1-(3,5-dimethylbenzyl)-1-benzo[d]imidazol-2-ylthio)acetamide (**5**)

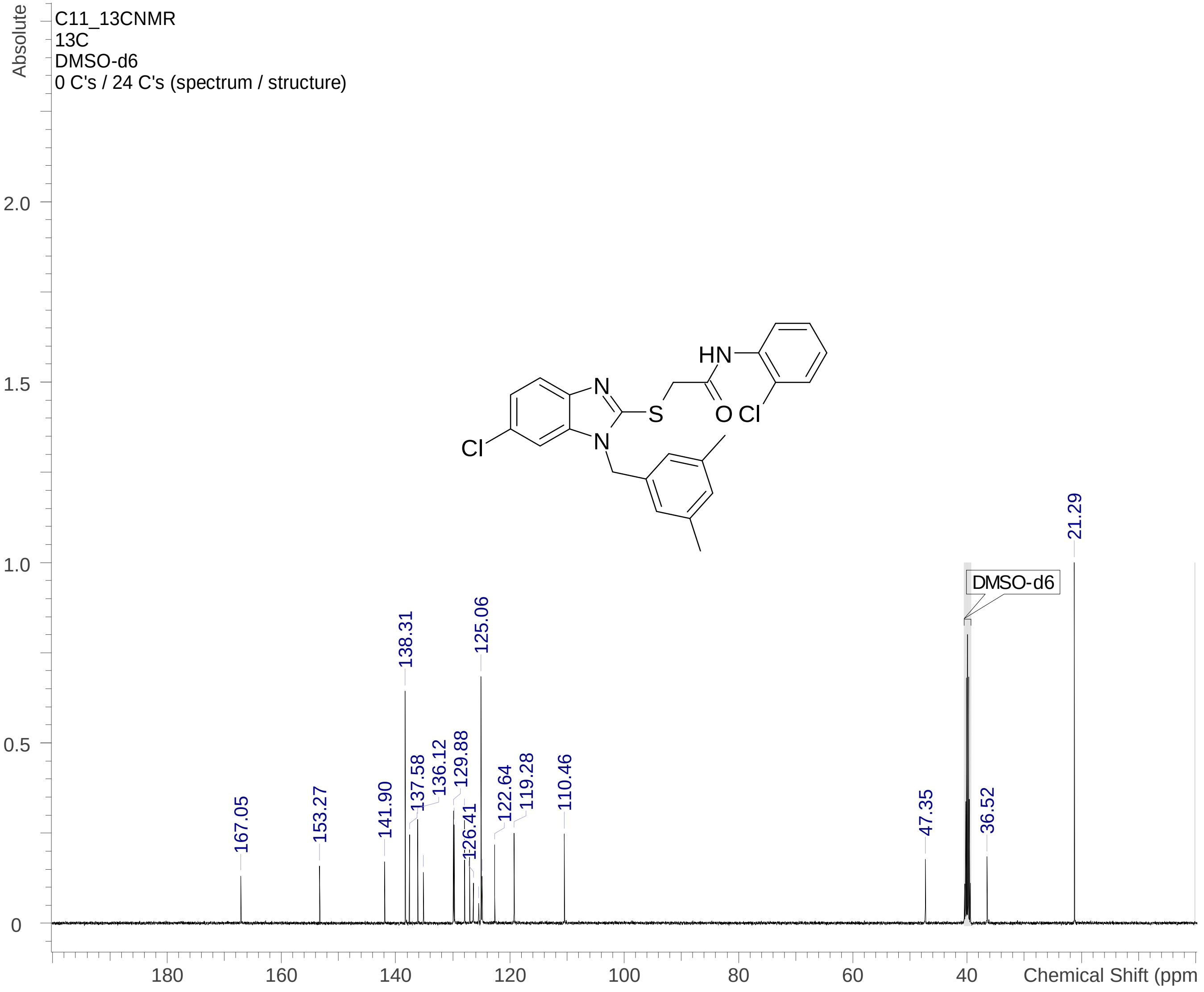

Figure S17: ^{13}C-NMR (DMSO-d_6) spectrum for 2-(6-chloro-1-(3,5-dimethylbenzyl)-1*H*-benzo[*d*]imidazol-2-ylthio)-*N*-(2-chlorophenyl)acetamide (**6**)

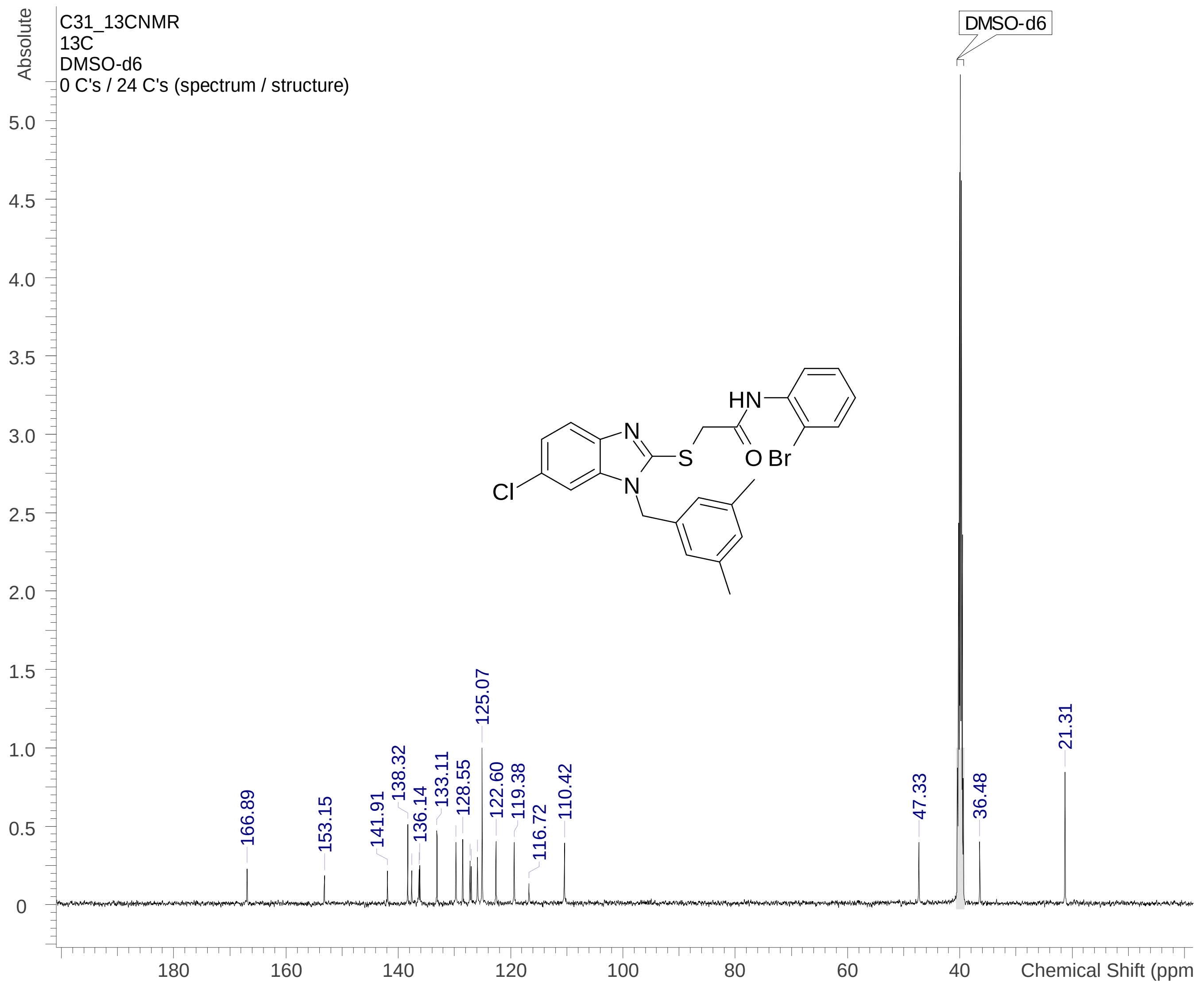

Figure S18: ^{13}C-NMR (DMSO-d$_6$) spectrum for *N*-(2-bromophenyl)-2-(6-chloro-1-(3,5-dimethylbenzyl)-1*H*-benzo[*d*]imidazol-2-ylthio)acetamide (**7**)

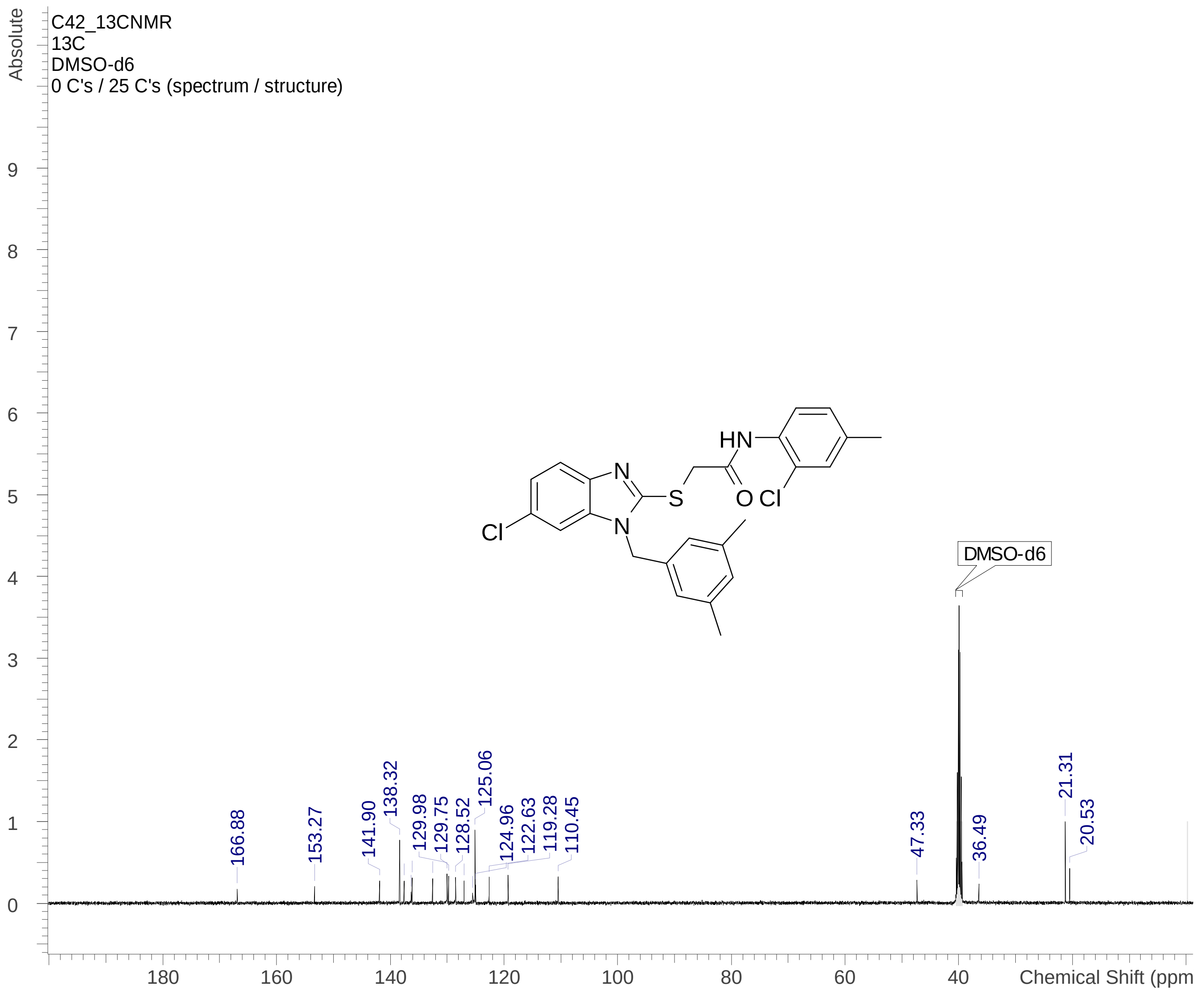

Figure S19: ^{13}C-NMR (DMSO-d_6) spectrum for 2-(6-chloro-1-(3,5-dimethylbenzyl)-1*H*-benzo[*d*]imidazol-2-ylthio)-*N*-(2-chloro-4-methylphenyl)acetamide (**8**)

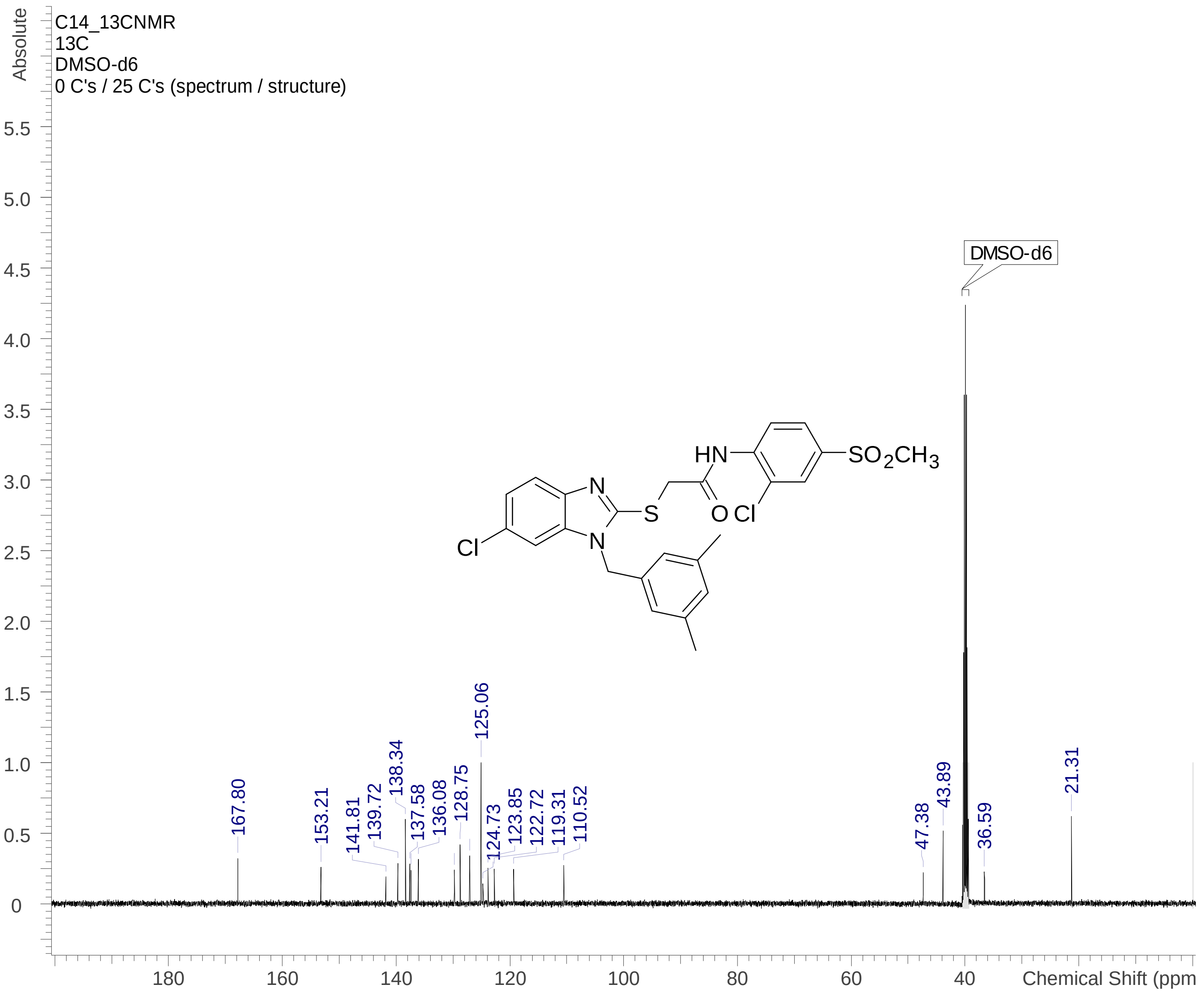

Figure S20: ^{13}C-NMR (DMSO-d_6) spectrum for 2-(6-chloro-1-(3,5-dimethylbenzyl)-1H-benzo[d]imidazol-2-ylthio)-N-(2-chloro-4-(methylsulfonyl)phenyl)acetamide (**9**)

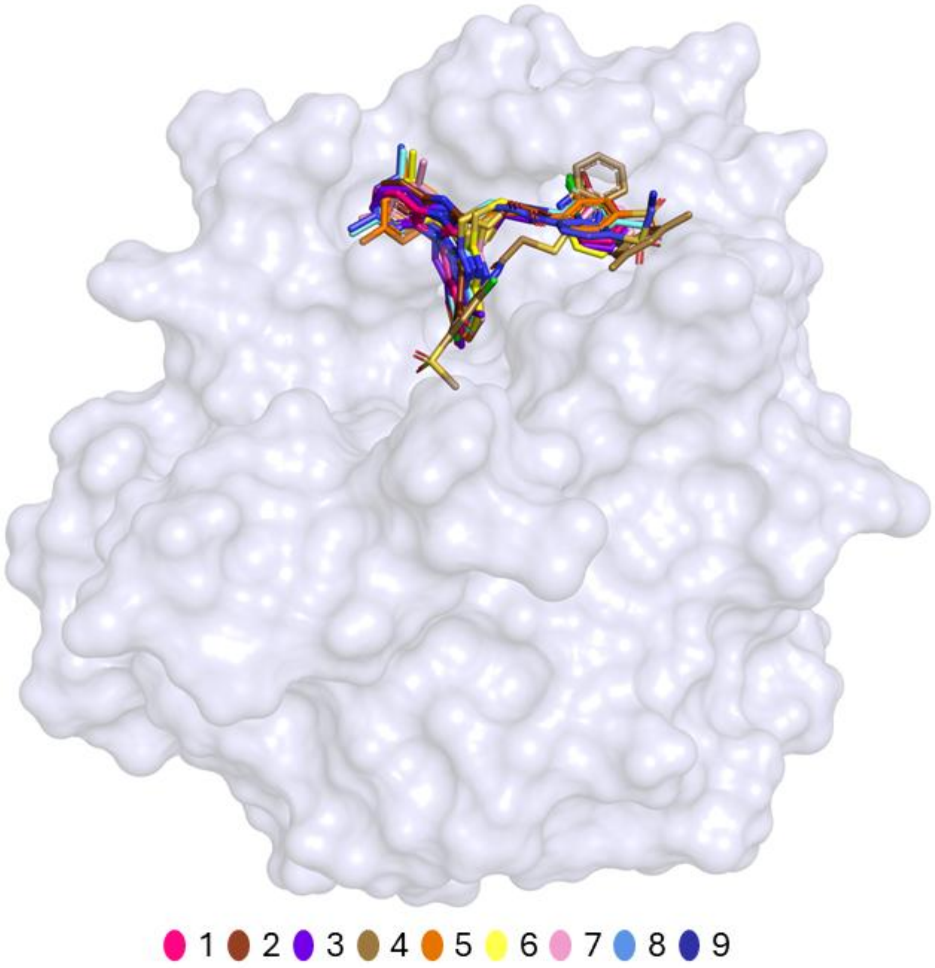

Figure S21: Overlay of the predicted binding poses of ligands **2–9**, represented as sticks in different colours, compared to the reference compound **1** (hotpink sticks) within the active site of porcine pancreatic elastase (PPE — PDB code: 1ELE). The protein structure is represented as lightblue surface. All ligands occupy a similar binding pocket, showing comparable orientation and interactions with key active site residues. The image is created with PyMOL. (https://www.pymol.org/)

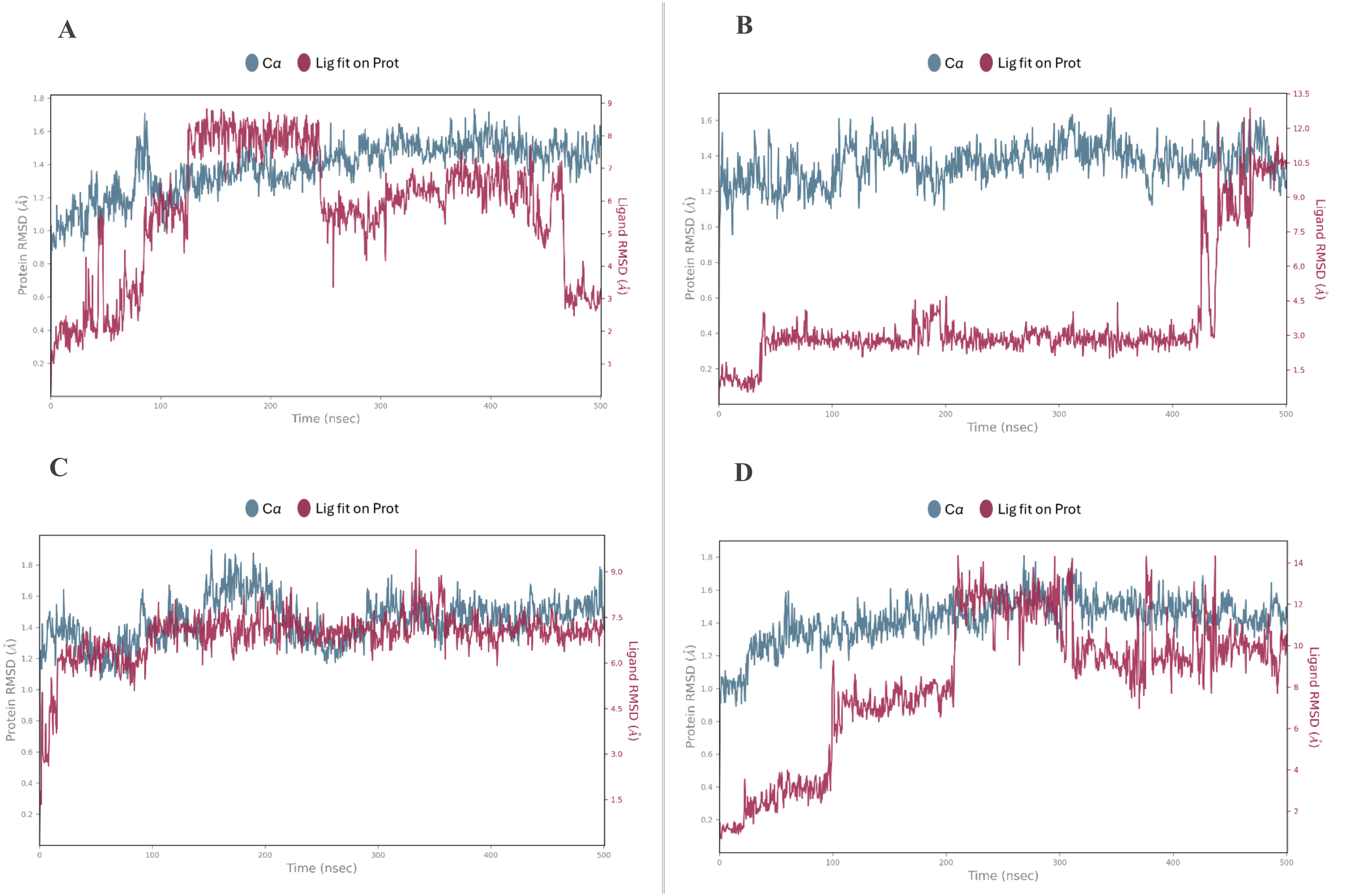
A
Cα
Lig fit on Prot
Protein RMSD (Å)
Ligand RMSD (Å)
Time (nsec)
B
Cα
Lig fit on Prot
Protein RMSD (Å)
Ligand RMSD (Å)
Time (nsec)
C
Cα
Lig fit on Prot
Protein RMSD (Å)
Ligand RMSD (Å)
Time (nsec)
D
Cα
Lig fit on Prot
Protein RMSD (Å)
Ligand RMSD (Å)
Time (nsec)

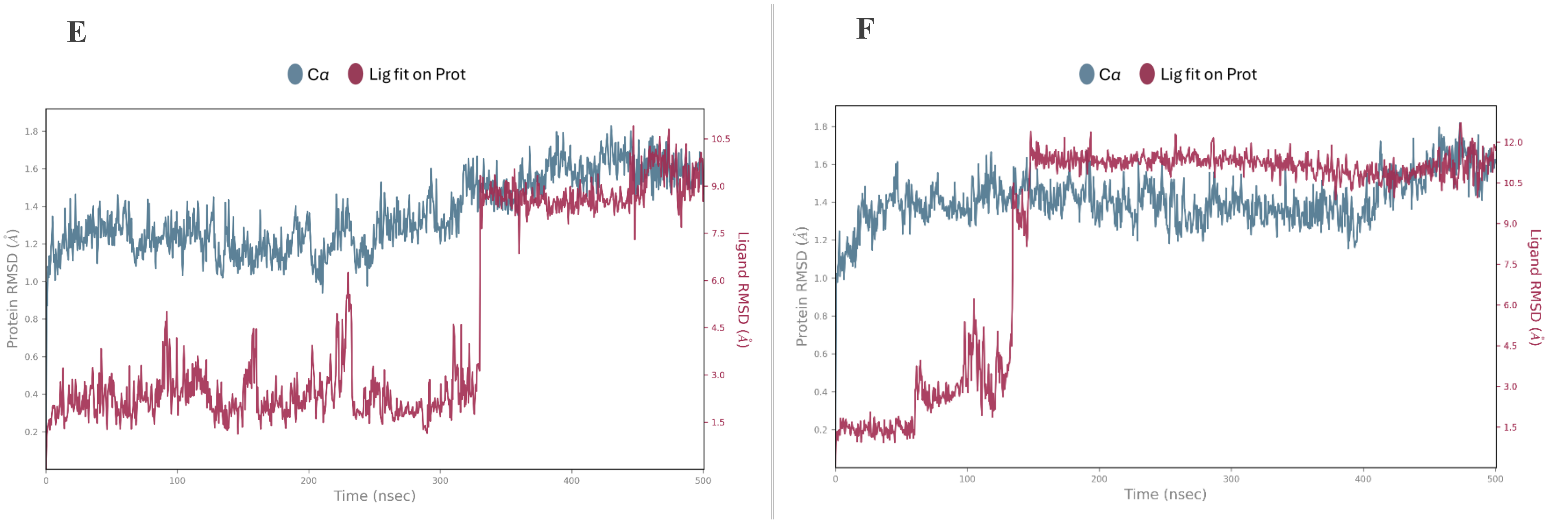

Figure S22: Individual RMSD plots for compounds **6** and **7** obtained from three independent 500 ns MD simulation replicas. Panels A, C, and E correspond to replicas 1, 2, and 3 of compound **6**, respectively, while panels B, D, and F show replicas 1, 2, and 3 of compound **7**.

Table S1: MM-GBSA results for all eight ligands (**2**-**9**) and reference compound **1**. The analysis was performed using the Prime MM-GBSA module of the Schrödinger Suite (Schrodinger 2025-3) on protein–ligand complexes that were previously minimized using the *Refine Protein–Ligand Complex* (Schrodinger 2025-3).

ID	MM-GBSA (Kcal/mol)
**1**	-105.11
**2**	-91.44
**3**	-101.83
**4**	-91.24
**5**	-97.89
**6**	-111.38
**7**	-112.34
**8**	-109.74
**9**	-103.95
